# Mental health in Central and Eastern Europe: a comprehensive analysis

**DOI:** 10.1016/j.lanepe.2025.101464

**Published:** 2025-10-06

**Authors:** Petr Winkler, Zoe Guerrero, Anna Kågström, Michaela Petrášová, Arlinda Cerga Pashoja, Gentiana Qirjako, Valentina Hristakeva, Dimitar Germanov, Martina Rojnic Kuzman, Dina Bošnjak Kuharić, Lucie Havlíková, Herman Eek, Eduard Maron, Dorottya Őri, Róbert Wernigg, Naim Fanaj, Elona Krasniqi, Liene Sile, Klinta Brinkmane, Karilė Levickaitė, Ugnė Grigaitė, Nensi Manusheva, Gjorgji Kalpak, Iva Ivanovic, Jana Chihai, Belous Mihaela, Tomasz M. Gondek, Agata Todzia-Kornaś, Adriana Mihai, Rebeca-Isabela Molnar, Katarína Molnárová Letovancová, Elena Kopcová, Orest Suvalo, Oleksandra Khudoba, Jamila Ismayilova, Gunel Muradova, Nino Makhashvili, Mishiko Dumbadze, Liliia Panteleeva, Mikhail Popkov, Lynn Al Tayara, Robert van Voren, Graham Thornicroft

**Affiliations:** a WHO Collaborating Centre for Public Mental Health Research and Service Development, National Institute of Mental Health, Topolová 748, Klecany 250 67, Czechia; b Centre for Global Mental Health, Health Service and Population Research Department, Institute of Psychiatry, Psychology and Neuroscience, King's College London, London, United Kingdom; c Department of Social Work, Faculty of Arts, Charles University, Na Příkopě 584/29, Prague, Czechia; d Global Public Health Department, Karolinska Institutet, Stockholm, Sweden; e St. Marys University, London, United Kingdom; f University of Medicine, Tirana, Albania; g Global Initiative on Psychiatry, Sofia, Bulgaria; h Zagreb School of Medicine and Zagreb University Hospital Centre, Zagreb, Croatia; i University Psychiatric Hospital Vrapce, Zagreb, Croatia; j Tallinn University of Technology, Tallinn, Estonia; k London Imperial College, London, United Kingdom; l Institute of Behavioural Sciences, Semmelweis University, Budapest, Hungary; m Department of Mental Health, Heim Pal National Pediatric Institute, Budapest, Hungary; n National Directorate-General for Hospitals, Budapest, Hungary; o Alma Mater Europaea Campus College Rezonanca, Pristina, Kosovo; p UBT Higher Education Institution, Pristina, Kosovo; q National Centre of Mental Health, Riga, Latvia; r Mental Health Perspectives, Vilnius, Lithuania; s Lisbon Institute of Global Mental Health, Comprehensive Health Research Centre, NOVA Medical School, Universidade NOVA de Lisboa, Lisbon, Portugal; t Faculty of Medicine, University “Ss. Cyril and Methodius” Skopje, North Macedonia; u University Psychiatry Clinic, Skopje, North Macedonia; v Center for Early Development, Clinical Centre of Montenegro, Podgorica, Montenegro; w Nicolae Testemitanu, State University of Medicine and Pharmacy, Chişinău, Moldova; x Institute of Social Studies, University of Lower Silesia, Wroclaw, Poland; y Military Institute of Medicine - National Research Institute in Warsaw, Poland; z University of Medicine, Pharmacy, Science and Technology George Emil Palade, Târgu Mureş, Romania; aa Department of Social Work, Faculty of Health Care and Social Work, Trnava University, Trnava, Slovakia; ab Tenenet o.z., Senec, Slovakia; ac Institute of Mental Health of Ukrainian Catholic University, Lviv, Ukraine; ad Institute of Public Administration, Governance and Professional Development of Lviv Polytechnic National University, Lviv, Ukraine; ae The National Mental Health Center of the Ministry of Health, Baku, Azerbaijan; af Ilia State University, Tbilisi, Georgia; ag Department of Medical Psychology, Psychiatry and Psychotherapy, Kyrgyz-Russian Slavic University, Bishkek, Kyrgyz Republic; ah Department of Propedotherapy of Family Medicine, International Higher School of Medicine, Bishkek, Kyrgyz Republic; ai World Health Organization, Barcelona Office for Health Systems Financing, Spain; aj Federation Global Initiative on Psychiatry, Hilversum, Netherlands; ak Vytautas Magnus University, Kaunas, Lithuania; al Department of Psychology, Faculty of Arts, Charles University, Prague, Czech Republic; am Department of Psychiatry, Psychosomatic Medicine, and Psychotherapy, University Hospital, Goethe University, Heinrich-Hoffmann-Straße 10, 60528 Frankfurt am Main, Germany

**Keywords:** Mental health, Central and Eastern Europe, Post-communist Europe, Children and young people, Migrants, Common mental disorders, Prevention, Promotion, Early detection, Early intervention

## Abstract

The post-communist WHO European region, often called Central and Eastern Europe (CEE), includes 28 countries with over 770 million people. Mental health systems remain shaped by the communist legacy of centralized institutions, a narrow biomedical focus, and neglect of social and psychological dimensions. Chronic underfunding persists, further strained by shrinking civic space in some countries and the war in Ukraine. Substantial progress has been made in the past decade, with modernization and rights-based approaches gaining ground. Yet reforms face entrenched barriers: underinvestment disproportionate to the burden; pervasive stigma, weak advocacy, and limited involvement of people with lived experience; dominance of institutional care over prevention, promotion, and community services; reliance on donor-driven projects that falter once funding ends; and human resource problems. Governance is often unstable, with low prioritization, clientelism, and personal biases undermining reforms. Research and data remain scarce, leaving systems unevaluated and vulnerable to reversal. Poor decision-making compounds these barriers: systemic missteps, driven by limited expertise, weak evidence, and personal biases, prevent resources from achieving the best possible outcomes. To move forward, CEE must integrate health, social, and education systems, secure sustainable crisis services, strengthen professional skills, involve people with lived experience, expand public mental health expertise, and, above all, commit greater and more transparent investment, closer to western European levels, if resilient and effective systems are to be built.

## Introduction

The post-communist WHO European region—commonly referred to as Central and Eastern Europe (CEE)—encompasses a culturally, socially, and economically diverse area. Excluding Russia, CEE comprises 28 countries grouped into six subregions, covering over 770 million people across 7.5 million km^2^: the Balkans (Albania, Bosnia and Herzegovina, Bulgaria, Croatia, Kosovo, Montenegro, North Macedonia, Romania, Serbia, and Slovenia), the Baltics (Estonia, Latvia, Lithuania), the Caucasus (Armenia, Azerbaijan, Georgia), Central Asia (Kazakhstan, Kyrgyzstan, Tajikistan, Turkmenistan, Uzbekistan), Central Europe (Czechia, Hungary, Poland, Slovakia), and Eastern Europe (Belarus, Moldova, Ukraine).

**Key messages**

• Central and Eastern Europe continues to experience high suicide rates, harmful alcohol use, and rising mental health burdens; particularly among youth and war-affected populations. These challenges are compounded by weak evidence-based planning, poor coordination, limited evaluation, and increasing strain from regional crises, such as the war in Ukraine, as well as global challenges like digital transformation and climate change.
• Mental health prevention and promotion in Central and Eastern Europe are widely recognized as policy priorities, with activities spanning schools, workplaces, primary care, social services, parenting programmes, and digital platforms; however, implementation remains uneven, underfunded, fragmented, and often NGO-driven, with limited evaluation, coordination, and evidence-based guidance.
• Reform momentum has increased, with new policies, expanded services, and support from European and international funding, but progress is uneven, and sustainability and scalability remain major gaps. Lasting improvement depends on political will, cross-sector collaboration, and better data to guide coordinated, evidence-driven action.

**Search strategy and selection criteria**

The literature search was conducted in the databases Ovid: PsycINFO, Global Health, Medline, and Embase. For prevention and promotion, the search strategy combined three concepts. The first concept—mental health—included the terms “mental health,” “mental disorder,” “mental illness,” “psychological disorders,” and “psychiatric disorders.” The second concept—prevention or promotion—included the indexed terms exp health promotion, exp primary prevention, exp secondary prevention, exp tertiary prevention, and exp prevention. The third concept—Central and Eastern Europe (CEE)—included the geographic terms “Central Europe,” “Eastern Europe,” “Central Eastern Europe,” “Albani∗,” “Bulgaria∗,” “Belarus∗,” “Bosnia and Herzegovin∗,” “Croatia∗,” “Czechia,” “Czech Republic,” “Estonia,” “Hungar∗,” “Kosov∗,” “Latvia∗,” “Lithuania∗,” “Macedonia∗,” “Montenegro∗,” “Moldov∗,” “Poland,” “Polish,” “Romania∗,” “Slovakia∗,” “Serbia,” “Slovenia∗,” “Ukrain∗,” “Armenia∗,” “Azerbaijan∗,” “Georgia∗,” “Kazak∗,” “Kazakhstan∗,” “Kyrgyzstan∗,” “Turkmenistan∗,” “Uzbek∗,” and “Central Asia.” These three concepts were combined using the Boolean operator AND. For early detection and intervention, a similar three-concept approach was applied. The first concept—mental health—used the same terms as above with the addition of “symptoms.” The second concept—early detection and intervention—included the indexed terms exp early detection, exp early intervention, exp screening, and exp case finding. The third concept—CEE—was identical to that used for prevention and promotion. These three concepts were also combined using AND. Inclusion criteria were systematic reviews, randomized controlled trials, controlled clinical trials, cohort studies, case-control studies, ecological studies, qualitative studies, and policy reports, with populations including adults, adolescents, and children in CEE countries. Interventions covered mental health promotion, prevention, early detection, and intervention programs targeting perinatal mental health, school, workplace, and digital settings; early intervention services for psychosis; mental health services in primary care and social care; and parenting programs. Eligible outcomes included mental health outcomes (such as depression, anxiety, psychosis, and suicide), quality of life, service utilization, cost-effectiveness, and implementation factors. Exclusion criteria were case reports, opinion papers, and studies focusing solely on pharmacological interventions without a broader mental health component. The grey literature search was conducted at the individual country level by the co-authors.

Secondary data on system characteristics were drawn from WHO’s Mental Health Atlas,^12^^,^^13^ the World Bank DataBank^54^ and the Global Burden of Disease database^55^ (all further referred to as ‘databases’).

Since the fall of communism, a range of crises has posed serious threats to population mental health across the region. These include the 1994 sinking of the MS Estonia, wars in the former Yugoslavia, Azerbaijan, Armenia, Georgia, Tajikistan, and Ukraine, ethnic conflicts in Kyrgyzstan, earthquakes in Albania and Croatia, and the environmental collapse of the Aral Sea—all of which have left deep, multi-generational psychological impacts.

Mental health systems across this region have been profoundly influenced by the 20th-century Soviet regime.^1^ This legacy, with few exceptions, is characterized by asylum-like psychiatric hospitals, institutionalization and centralization, a biomedical orientation, marginalization of social and psychological aspects of health, and an authoritarian approach to clinical decision-making.^2^ Systemic deficiencies include weak public health infrastructure and expertise, non-transparent decision-making, human rights violations, and low mental health literacy including pervasive stigma.^3^ While brain drain constitutes a substantial challenge to the region^4^ many countries maintain relatively high numbers of psychiatrists and other mental health professionals^5^ supported by robust social security systems–despite many countries in the region being classified as low or middle-income.

Since the fall of the Berlin wall, mental health systems across the region have undergone significant transformation. In Central Asia, mental health services collapsed in the 1990s and have only recently begun to recover.^6^ Elsewhere, countries have transitioned from centrally governed, state-funded systems to insurance-based models with progress in legislation and increased recognition and protection of the rights of people with mental illness.^7^^,^^8^ Training for young professionals has improved.^8^ Yet, as of 2016, care of people with severe mental illness—particularly the process of deinstitutionalization—remained limited, and government-led reforms unimplemented.^3^^,^^5^ Consequently, key challenges persisted, more than two decades after the dissolution of the USSR.^3^

Currently, mental health systems in the region face rising demands alongside major global trends, including digital transformation and climate change^9^, ^10^, ^11^ as well as region-specific challenges, most notably the ongoing war in Ukraine. Scalable investments are critical to address these challenges and improve population outcomes. Against this backdrop, we assessed the current state of the field across the region, with a special focus on promotion, prevention and early intervention.

## Methods

This study aimed to map and analyze mental health care systems to understand mental health promotion, prevention, early detection, and early intervention across post-communist countries in the WHO CEE region, excluding Russia. Countries included cover five subregions: Balkans, Baltics, Caucasus, Central Asia, Central Europe, and Eastern Europe. We applied a dual-method approach: a systematic scoping review and a mixed-methods, multi-country scoping review (see Appendix for in depth description of methods employed). The systematic review followed PRISMA guidelines, included two search streams and used predefined inclusion criteria. Data were independently screened and extracted by two reviewers (ZG, MP), supplemented by secondary data from WHO, World Bank, and GBD databases.

The multi-country scoping review combined structured expert led literature scans with key informant interviews conducted in local languages. Over 115 expert interviews were conducted across 18 countries, ranging from 3 interviews in Georgia to 24 in Moldova. A slightly higher proportion of females were interviewed (62% females). Interviewees represented a broad range of roles, including psychiatrists, child and adolescent mental health professionals, psychologists, social workers, public health officials, NGO representatives, healthcare managers, academics, migration and policy experts, and individuals with lived experience of mental health conditions.

Collaborators used a standardized questionnaire based on the WHO Mental Health Care Pyramid to report on various care levels and thematic areas, including child and adolescent mental health, migrant mental health, and common mental disorders. Two framework analyses synthesized findings: one focused on mental health systems across six domains (policy and governance, funding and resources, service delivery and access, workforce and training, social and cultural context, and monitoring, evaluation, and research); the other on prevention and early intervention across eight domains (policy and planning, primary care, schools, self-care, targeted interventions, funding, evaluation, and early detection and intervention). Coding was performed by two reviewers (PW, AK) using iterative codebook development, and findings were triangulated across data sources (see more details on methods in the Appendix).

## Burden of mental ill health

**Fig. 1:**
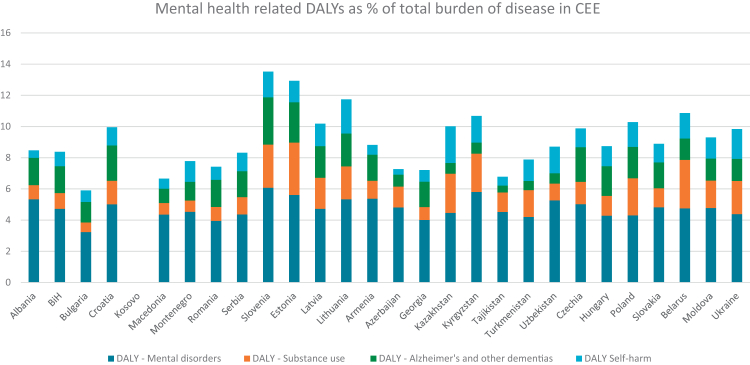
**Mental health related disability adjusted life years expressed as a percentage of total burden of disease in Central and Eastern Europe**. Based on data from the Institute for Health Metrics and Evaluation: Mental health related DALY (disability adjusted life years) 2021.

With the exception of Muslim-majority countries, the region has relatively high rates of suicides—reaching 20.1 per 100,000 in Latvia—and high alcohol consumption—up to 17.5 L of pure alcohol per capita (aged 15+) annually. Mental disorders account for a substantial share of disease burden, ranging from 7.8% of total DALYs in the Caucasus to 11.6% in the Baltics (see Table 1, Fig. 1 and Appendix).

**Table 1:** Characteristics of individual countries and subregions of CEE as compared to other EU 14 countries.

Sub-region	Country	Pop. (000 persons)	Surface area (sq. km)	GDP per capita PPP	Life expect	Alcohol cons. per capita (l)	Suicides per 100 k pop.	MH DALY share on total (%)	MH share on health exp. (%)	Refugees from Ukraine per 100 k pop.
Balkans	Albania	2745.97	28,750	21,208	77	4.5	3.7	8.5	NA	235
Balkans	BiH	3185.07	51,210	22,391	75	5.9	8.3	8.4	NA	9
Balkans	Bulgaria	6446.6	111,000	37,411	74	11.6	6.5	5.9	2.6	1200
Balkans	Croatia	3859.69	88,070	45,485	78	7.7	14.1	10.0	NA	719
Balkans	Kosovo	1756.37	10,887	15,141	80	NA	NA	NA	NA	NA
Balkans	Macedonia	1827.82	25,710	24,327	74	4.4	7.2	6.7	7.3	1057
Balkans	Montenegro	616.18	13,810	30,887	76	17.5	16.2	7.8	NA	3062
Balkans	Romania	19059.48	238,400	45,659	75	NA	16.8	7.4	NA	943
Balkans	Serbia	6623.18	84,990	28,674	75	7.9	7.9	8.3	NA	164
Balkans	Slovenia	2120.46	20,480	53,813	81	10.4	14.0	13.5	NA	620
**Balkans**	**Total**/Average	48240.82	673,307	32,500	77	7.8	10.5	8.5	–	197
Baltics	Estonia	1370.29	45,340	46,669	78	10.7	12.0	12.9	3.7	3105
Baltics	Latvia	1877.44	64,590	41,384	75	12.9	16.1	10.2	4.8	2586
Baltics	Lithuania	2871.59	65,286	50,783	76	12.1	20.2	11.7	4.2	1695
**Baltics**	**Total**/Average	6119.3	175,216	46,279	76	11.9	16.1	11.6	4.2	2462
Caucasus	Armenia	2990.9	29,740	21,343	73	4.3	2.7	8.8	2.3	20
Caucasus	Azerbaijan	10153.96	86,600	23,598	73	2.5	4.0	7.3	3.5	48
Caucasus	Georgia	3715.48	69,700	25,072	72	14.4	8.2	7.2	1.9	802
**Caucasus**	**Total/**Average	16860.34	186,040	23,337	73	7.1	4.9	7.8	2.6	290
C Asia	Kazakhstan	20330.1	2,724,902	38,515	74	4.5	18.1	10.0	NA	NA
C Asia	Kyrgyzstan	7099.75	199,950	7107	72	3.6	NA	10.7	NA	NA
C Asia	Tadjikistan	10389.8	141,379	4964	71	0.7	NA	6.8	NA	NA
C Asia	Turkmenistan	7364.44	491,209	19,829	69	2.6	6.1	7.9	NA	NA
C Asia	Uzbekistan	35652.31	448,924	11,107	72	2.1	NA	8.7	NA	NA
**C Asia**	**Total**/Average	126878.5	4,596,667	19,518	100	3.3	–	8.8	–	NA
C Europe	Czechia	10864.04	78,871	53,080	79	12.0	13.1	9.9	4.0	3669
C Europe	Hungary	9592.19	93,030	44,905	76	9.9	11.8	8.7	3.2	650
C Europe	Poland	36687.35	312,720	46,450	77	11.7	9.3	10.3	3.5	2720
C Europe	Slovakia	5426.74	49,030	43,513	77	10.7	12.8	8.9	NA	2447
**C Europe**	**Total**/Average	235490.9	533,651	46,987	77	11.1	11.7	9.4	3.6	3162
E Europe	Belarus	9178.3	207,630	30,763	73	11.6	16.5	10.9	1.9	NA
E Europe	Moldova	2457.78	33,850	17,597	69	11.1	12.2	9.3	2.5	5199
E Europe	Ukraine	37732.84	603,550	17,630	69	9.2	17.7	9.8	NA	NA
**E Europe**	**Total**/Average	336566.1	1,378,681	28,244	72	10.7	15.5	9.9	2.2	–
**CEE**	**Total**/Average	770156.1	7,543,562	31,047	75	8.3	11.7	9.1	3.2	1528
**EU 14**	**Total**/Average	346730.5	3,078,153	72,096	82	9.5	8.6	15.9	8.7	769

Data for individual countries are taken from the following sources: 1) World Bank: population (2023), surface area (2023), GDP per capita in current international USD and purchasing power parity (2023), life expectancy (2023), total alcohol consumption per capita (liters of pure alcohol, projected estimates, 15+ years of age; 2020); 2) WHO Mental Health Atlas (MHA) 2020: suicide rates per 100,000 population, the government’s total expenditure on mental health as % of total government health expenditure–for Czechia, Slovakia and Georgia the data come from WHO MHA 2017; 3) Institute for Health Metrics and Evaluation: Mental health related DALY (disability adjusted life years) 2021—calculated as a sum of DALY for mental disorders, substance use, self-harm, and Alzheimer’s disease and other dementias (see Appendix for details); 4) UNHCR–Situation Ukraine Refugee Situation–Operational Data Portal: number of refugees from Ukraine as per 20 March 2025 (number of refugees in Serbia includes refugees in Kosovo as well). EU 14 countries include Austria, Belgium, Denmark, Finland, France, Germany, Greece, Ireland, Italy, Luxembourg, Netherlands, Portugal, Spain, and Sweden.

pop.—population, sq. km—square kilometres, GDP—gross domestic product, PPP—purchasing power parity, expect.—expectancy, k—thousands, MH—mental health, DALY—disability adjusted life years, cons.—consumption, exp.—expenditures, C—central, E—Eastern.

## Mental health care systems

### Policy and governance

Mental health systems in CEE remain heavily reliant on institutional care reflected in a high number of beds in psychiatric hospitals per capita and disproportionate share of government spending on psychiatric hospitals. Outpatient service availability for both adults and young people varies widely across the region. In many contexts, service models are shaped by the dominance of psychiatry and professional privatization rather than holistic or rights-based approaches, with multi-disciplinary, community care driven by bottom-up or international initiatives, at best in collaboration with local governments. According to WHO MHA data, integration of mental health into primary care is partial but progressing and most countries have adopted mental health strategies guiding reform. These policies often emphasize deinstitutionalization while addressing primary care integration, stakeholder collaboration, facility conditions, and promotion and prevention. The current reform efforts aim to establish an integrated, intersectoral model that ensures improved quality of life and upholds human rights and freedoms. See Table 2 for country specific indicators of mental health systems.

**Table 2:** Basic characteristics of mental health care systems in CEE.

Sub-region	Country	MH plan	MH plan year	Suicide plan	Integr. into PHC	Total outpat. facilities	Total outpat. CAMH f.	Mental hospitals	Beds in MH per 100 k	Exp. to ment. hosp. (%)
Balkans	Albania	Yes	2013	No	3	14	6	2	16.31	NA
Balkans	BiH	Yes	2012	No	3	792	1	4	14.72	NA
Balkans	Bulgaria	No	–	No	2	69	NA	12	30.81	34
Balkans	Croatia	Yes	2022	Yes	4	1354	24	8	75.73	NA
Balkans	Kosovo	NA	NA	NA	NA	NA	NA	NA	NA	NA
Balkans	Macedonia	Yes	2018	No	1	52	5	4	93.59	19.8
Balkans	Montenegro	Yes	2019	No	0	4	NA	2	44.75	NA
Balkans	Romania^a^	Yes	2009	NR	NR	173	63	34	82.47	NA
Balkans	Serbia	Yes	2019	No	3	4	47	7	48.71	NA
Balkans	Slovenia	Yes	2018	Yes	5	128	683	5	55.95	NA
**Balkans**	**Total**/Average	–	–	–	2.6	2590	829	78	51.4	–
Baltics	Estonia	No	–	No	5	NA	12	2	8.67	NA
Baltics	Latvia	Yes	2019	No	5	NA	276	5	111.18	78.3
Baltics	Lithuania	Yes	2020	Yes	4	196	NA	6	41.71	7.9
**Baltics**	**Total**/Average	–	–	–	4.7	–	288	13	53.9	43.1
Caucasus	Armenia	Yes	2014	No	2	NA	1	9	41.25	88.6
Caucasus	Azerbaijan	Yes	2012	No	5	22	NA	11	38.08	91.5
Caucasus	Georgia^a^	Yes	2014	NR	NR	NA	12	9	36.72	NA
**Caucasus**	**Total**/Average	–	–	–	3.5	–	13	29	38.7	90.1
C Asia	Kazakhstan	Yes	2020	No	4	63	10	19	42.72	NA
C Asia	Kyrgyzstan	NA	NA	NA	NA	NA	NA	NA	NA	NA
C Asia	Tajikistan	Yes	2018	No	3	NA	NA	8	27.52	NA
C Asia	Turkmenistan	NA	NA	NA	NA	NA	NA	NA	NA	NA
C Asia	Uzbekistan	NA	NA	NA	NA	NA	NA	NA	NA	NA
**C Asia**	**Total**/Average	–	–	–	3.5	–	–	–	–	–
C Europe	Czechia	Yes	2020	Yes	4	1238	NA	23	81.24	55.7
C Europe	Hungary	Yes	2018	Yes	2	1155	97	1	3.21	35.1
C Europe	Poland	Yes	2017	Yes	NA	2170	340	46	28.58	30.4
C Europe	Slovakia^a^	Yes	2017	No	NA	NA	NA	NA	NA	NA
**C Europe**	**Total**/Average	–	–	–	3	4563	–	70	37.7	40.4
E Europe	Belarus	Yes	2016	Yes	4	107	66	20	60.5	81.8
E Europe	Moldova	Yes	2017	No	3	41	NA	3	35	84.8
E Europe	Ukraine	Yes	2017	No	3	658	555	60	61.05	NA
**C Europe**	**Total**/Average	–	–	–	3.3	806	621	83	52.2	83.3
**CEE**	**Total**/Average	–	–	–	3.4	7959	1751	273	46.8	64.2

Data based on WHO Mental Health Atlas 2020.

integr.—integration, outpat.—outpatient, k—thousands, exp.—expenditures, ment. hosp.—mental hospitals, C—central, E—Eastern.

a Data based on WHO MHA 2017; MH Plan Implementation based on country reports compiled by co-authors representing the respective countries (NI = Not implemented; II = implementation initiated; PI = partially implemented; IS = implemented on a scale); comparison with EU 14 not conducted—data not reliable. Data in italics are corrections made by country experts, not from the databases.

Implementation of reforms varies significantly across the region. Poland has successfully developed an extensive network of community mental health services that integrate medical and social care. Other countries, such as Georgia, Czechia, and Ukraine, have established several such services, whereas some, such as Slovakia, have yet to begin implementation.

Mental health care governance remains highly fragmented. While ministries of health oversee clinical services, ministries of social and educational affairs cover complementary services, with limited coordination. Non-governmental organizations (NGOs) play an essential role, particularly in prevention and promotion, areas pervasively neglected by states. While countries like Czechia, Slovakia, and Ukraine have established interministerial platforms, and Bulgaria approved a cross-sectoral collaborative Framework Agreement in 2006 between the Ministry of Health and Ministry of Labor and Social Policy, weak intersectoral collaboration remains a major barrier to system-wide progress in mental health care.

### Funding and resources

Despite high rates of suicides, elevated alcohol consumption and a growing burden of mental disorders, countries in CEE, invest on average only 3.2% of total government health expenditures to mental health (Table 1) with most directed to psychiatric hospitals (Table 2). International donors—including the EU, WHO, and others have provided critical support to initiate reform and respond to regional needs. However, reliance on external funding poses risks to stability, sustainability and scalability. This vulnerability is increasingly evident in several countries, particularly Ukraine, where the partial dissolution of USAID, a significant funder of mental health transformation, has raised concerns about the durability of reforms.

### Service delivery and accessibility

With few exceptions, systems in CEE remains predominantly reliant on hospitals, with outpatient services networks providing pharmacological treatments by psychiatrists and limited psychosocial care. According to expert sources, in some countries, such as Slovakia, Hungary and Estonia, high hospitalization rates are reported as due to a lack of community-based alternatives. The observation of high hospitalization rates occurring due to a lack of community-based alternatives warrants further exploration within the region.

Most countries have developed a wide network of social services ranging from crisis centres for children affected by violence, abuse or trafficking, to targeted services for homeless populations, migrants or socially excluded communities, and senior homes and respite care for people with dementia. These services though remain poorly integrated with mental health care despite the populations being at elevated risk for needing mental health support. Staff are frequently undertrained in recognizing and managing mental health problems, and there is little structured effort to incorporate these services systematically and generate structural pathways within multi-sectoral comprehensive networks for mental health care.

The COVID-19 pandemic served as a catalyst for mental health care development in the region, heightening public and political awareness of mental health issues and accelerating the adoption of telepsychiatry in several countries. While this resulted in better access to care, these advances have not been translated into sustained system-wide improvements.

Digital mental health resources are increasingly available in local languages and target diverse groups, from perinatal mental health to cognitive decline and suicide bereavement. Initiatives range from government-led campaigns to NGO-led projects. Despite their promise, many lack a strong theoretical foundation and robust evaluation. Systematic evaluation and evidence-informed design would greatly enhance their effectiveness and contribution to mental health promotion in the region.

### Workforce and training

Data from the WHO Mental Health Atlas highlight substantial disparities in the availability of several mental health professionals in the region. The Baltics and Central Europe report an average of 16.2 and 13.3 psychiatrists per 100,000 population respectively, which is about the same as the average in western EU countries (13.3 per 100,000 population). In Caucasus and Central Asia the number is much lower—3.9 and 2.9 per 100,000 population respectively. However, the data shows that the number of child psychiatrist is low across the region (3.9 per 100,000 population), the only exceptions being Lithuania (9.92), Hungary (9.66), Estonia (8.62) and Slovenia (7.65). Similarly, the availability of other key professionals—mental health nurses, psychologists, and social workers—is generally limited (see Table 3 for details).

**Table 3:** Number of mental health professionals across the region.

Sub-region	Country	Psychiatrists		MH Nurses		Psychologists		Social workers		Tot. workforce		CAMH psychiatr.		CAMH workforce	
		Nr.	Per 100 k	Nr.	Per 100 k	Nr.	Per 100 k	Nr.	Per 100 k	Nr.	Per 100 k	Nr.	Per 100 k	Nr.	Per 100 k
Balkans	Albania	46	1.6	250	8.68	43	1.49	34	1.18	393	13.64	8	1.13	60	8.46
Balkans	BiH	313	9.48	872	26.42	122	3.7	61	1.85	1478	44.77	1	0.15	180	27.36
Balkans	Bulgaria	695	9.93	978	13.97	89	1.27	50	0.71	1842	26.31	46	3.45	46	3.45
Balkans	Croatia	527	12.81	1831	44.33	234	5.67	55	1.33	2802	67.84	53	6.6	289	35.96
Balkans	Kosovo	NA	NA	NA	NA	NA	NA	NA	NA	NA	NA	NA	NA	NA	NA
Balkans	Macedonia	179	8.59	376	18.05	88	4.22	13	0.62	659	31.63	8	1.72	108	23.25
Balkans	Montenegro	55	8.76	NA	NA	28	4.46	13	2.07	109	17.36	NA	NA	NA	NA
Balkans	Romania	1125	5.66	3719	18.71	294	1.48	99	0.5	5351	26.92	112	0.56	NA	NA
Balkans	Serbia	492	5.61	1875	21.37	NA	NA	NA	NA	2367	26.98	47	2.5	77	4.1
Balkans	Slovenia	254	12.22	820	39.45	251	12.08	40	1.92	1583	76.16	31	7.65	121	29.87
**Balkans**	**Total**/Average	3686	8.3	10,721	23.9	1149	4.3	365	1.3	11,940	36.8	306	3.0	881	18.9
Baltics	Estonia	208	15.69	289	21.8	275	20.74	81	6.11	929	70.08	24	8.62	24	8.62
Baltics	Latvia	249	13.06	542	28.43	601	31.52	3315	173.86	5408	283.63	20	5.08	1237	313.92
Baltics	Lithuania	544	19.71	1034	37.47	523	18.95	549	19.89	2660	96.39	54	9.92	1824	334.98
**Baltics**	**Total**/Average	1001	16.2	1865	29.2	1399	23.7	3945	66.6	8997	150.0	98	7.9	3085	219.2
Caucasus	Armenia	50	1.69	278	9.4	50	1.69	NA	NA	378	12.78	14	1.79	96	12.26
Caucasus	Azerbaijan	336	3.34	720	7.17	138	1.37	50	0.5	1430	14.23	41	1.37	375	12.51
Caucasus	Georgia	265	6.71	NA	NA	92	2.33	NA	NA	369	9.34	12	0.3	NA	NA
**Caucasus**	**Total**/Average	651	3.9	998	8.3	280	1.8	50	0.5	2177	12.1	67	1.2	471	12.4
C Asia	Kazakhstan	803	4.33	3312	17.85	262	1.41	62	0.33	4476	24.13	184	2.86	778	12.1
C Asia	Kyrgyzstan	NA	NA	NA	NA	NA	NA	NA	NA	NA	NA	NA	NA	NA	NA
C Asia	Tajikistan	187	2.01	261	2.8	NA	NA	NA	NA	454	4.87	6	0.14	21	0.49
C Asia	Turkmenistan	134	2.26	NA	NA	1	0.02	0	0	135	2.27	8	0.35	10	0.43
C Asia	Uzbekistan	NA	NA	NA	NA	NA	NA	NA	NA	NA	NA	NA	NA	NA	NA
**C Asia**	**Total**/Average	1124	2.9	3573	10.3	263	0.7	62	0.2	5065	10.4	198	1.1	809	4.3
C Europe	Czechia	1668	15.6	3248	30.39	359	3.35	120	1.12	5644	52.8	NA	NA	NA	NA
C Europe	Hungary	1170	12.08	1052	10.86	1535	15.85	NA	NA	4023	41.54	182	9.66	1963	104.15
C Europe	Poland	4589	12.11	11,189	29.53	4620	12.19	NA	NA	25,334	66.87	360	4.8	3340	44.56
C Europe	Slovakia	NA	NA	NA	NA	NA	NA	NA	NA	NA	NA	NA	NA	NA	NA
**C Europe**	**Total**/Average	7427	13.3	15,489	23.6	6514	10.5	120	1.1	35,001	53.7	542	7.2	5303	74.4
E Europe	Belarus	1065	11.27	NA	NA	506	5.35	34	0.36	1899	20.09	103	5.04	103	5.04
E Europe	Moldova	136	3.36	542	13.41	52	1.29	58	1.43	848	20.97	8	0.94	105	12.38
E Europe	Ukraine	2463	5.6	10,551	23.98	412	0.94	305	0.69	15,104	34.33	245	2.73	343	3.82
**E Europe**	**Total**/Average	2599	6.7	11,093	18.7	970	2.5	397	0.8	17,851	25.1	356	2.9	551	7.1
**CEE**	**Total**/Average	16,488	8.5	43,739	19.0	10,575	7.3	4939	11.8	81,031	48.0	1567	3.9	11,100	56.0
**EU 14**	Average	–	13.3	–	45.8	–	41.4	–	17.6	–	141.2	–	11.8	–	61.0

The data are taken from the WHO Mental Health Atlas 2020. The data for Romania are from the WHO Mental Health Atlas 2017 since the 2020 country report was not available. The data for EU 14 countries are based on country reports from Finland, France, Germany, Ireland, Italy, Portugal, Spain, and Sweden since country reports for other countries were not available or did not contain relevant data.

nr.—number, k—thousands, tot.—total, MH—mental health, CAMH—child and adolescent mental health, psychiatry.–psychiatrists, C—central, E—Eastern.

Country-level reports highlight workforce shortages of qualified mental health professionals, particularly in child and adolescent mental health services, which are overwhelmed by surging need. Rising mental health needs amongst adults further strain limited resources. Access is particularly restricted in rural and remote areas, for example in Croatia, island residents must travel considerable distances for care, highlighting stark geographic inequities in service provision.

### Devastating impact of Russia’s war against Ukraine

Russia’s military aggression against Ukraine has a devastating impact on the mental health of its population within and beyond Ukraine’s border. More than a decade of hostilities, including over three years of full-scale war, has led to immense civilian suffering and humanitarian crisis. Continued attacks on populated areas have destroyed vital infrastructure, including health facilities. Since August 2023, intensified violence in regions such Kharkiv, Sumy, Zaporizhzhia, Kherson and Donetsk have triggered further evacuations and prolonged displacement. The cumulative impact of Russia’s war and continuous exposure to psychological dangers contributes to rising psychological distress, trauma, anxiety, and depression.

As of August 2024, approximately 6.7 million Ukrainians had fled the country, and 3.7 million remained internally displaced, many with limited access to essential health services, particularly in front-line and border regions. Among Ukrainians living abroad, high levels of mental health problems have been reported, with concerns that these will magnify with prolonged conflict.

Despite notable reforms and resilience, the mental health burden resulting from the war is vast. In CEE countries hosting large numbers of refugees (see Table 1), systems are unprepared, and needs are still emerging. These countries have not historically faced such immense migration fluxes and integration, and mental health and psychosocial support systems are being developed ad hoc to meet need. Many struggle to integrate qualified Ukrainian mental health professionals into their national workforce effectively, again leaving non-governmental organizations to fill critical gaps— raising concerns about sustainability.

### Influence of international and non-governmental organizations

International organizations such as the World Health Organization (WHO), the European Union (EU), and UNICEF and numerous non-governmental and volunteer organizations have been critical in supporting, mental health system development in the region. EU and UN agencies have been also instrumental in shaping national and regional response to the large influx of refugees by funding and coordinating mental health and psychosocial support (MHPSS). Such efforts span self-help interventions, help-lines psychosocial care, and capacity-building for teachers working with displaced families. Still, the scale of, migrants has overwhelmed mental health systems in CEE (see Table 1) and intense pressures continue to challenge local mental health services, including crisis centres, and outpatient and inpatient mental health services.

### Monitoring, evaluation and research

Despite significant efforts to develop mental health care over the past decade, nearly all CEE country experts report a lack of systematic monitoring and evaluation. Existing assessments are typically limited to project-specific evaluations, often linked to externally funded initiatives, such as the European Structural and Investment Funds (ESIF) and rarely assess long-term impact, effectiveness, or cost-effectiveness.

National government-led oversight authorities tasked with monitoring mental health services exist yet are limited to focus on compliance with standards and regulations rather than generating robust evidence to inform policy and system development. The striking paucity of published evidence aligns with expert validation, with nearly all highlighting a general absence of evaluation activity across the region, though more research is needed to uncover localized understandings of good practice and evaluation cultures. Rare exemptions exist, for example, Estonia has implemented a national Health System Performance Assessment (HSPA) framework developed with OECD support, that goes beyond project monitoring. In Moldova, international donor supported initiatives have successfully carried out impact evaluations of interventions. Poland and Czechia have also invested in generating evidence for informed decision-making, though the extent of its practical use varies. Several countries, including Bulgaria, Czechia, Croatia, Hungary and Slovakia, operate national centres with routinely collect health service data that could support epidemiological research and long-term systematic and comprehensive national, yet these are underutilized for evidence-based decision making.

Despite some alignment with evidence, critical gaps persist. In some cases, decision-making has disregarded established evidence, such as Czechia’s exemption of non-sparkling wines from excise duty. While there are nationwide initiatives, such as Croatia’s program aimed at supporting child and adolescent mental health, which has been gradually implemented in schools across the country, such programmes have not undergone rigorous evaluation that are peer reviewed and published. Evidence-based programmes—such as Housing First, Individual Placement and Support, and youth-oriented models remain largely absent across the region.

## Prevention, promotion, early detection and early intervention

Findings from literature The review of existing literature presents a fragmented overview of mental health prevention, promotion, early detection and early intervention. According to the WHO Mental Health Atlas 2020, progress in mental health promotion, prevention, and early detection remains limited across CEE.^12^ Fewer than half of the countries reported implementing at least two functioning prevention or promotion programmes, showing little improvement since 2017.^13^ While some integration into primary health care has occurred, access to community-based services remains uneven, and there are persistent gaps in early detection capacities.

An analysis of 33 studies focused on mental health promotion and prevention across the region found efforts targeting diverse populations–including adolescents,^14^, ^15^, ^16^, ^17^, ^18^, ^19^ parents,^15^^,^^20^^,^^21^ healthcare professionals and the public^22^, ^23^, ^24^, ^25^–through both digital^17^^,^^26^ and in-person methods especially school settings.^27^, ^28^, ^29^ Epidemiological studies examined risk and protective factors, with a particular focus on perinatal mental health,^30^^,^^31^ suicide,^32^^,^^33^ and social determinants.^34^ Suicide-related research showed mixed trends, with improvements in some countries and persistent challenges in others.^19^ Qualitative studies also highlighted the role of stigma, rurality, and service gaps in suicide risk.^34^

Several youth- and school-based prevention initiatives were evaluated, such as Romania’s SCHOLARS program^35^ and parenting support interventions in North Macedonia and Moldova.^20^ Digital strategies and community-based approaches played a significant role, with long-standing online platforms in Slovenia^17^ and multi-country suicide prevention programmes showing positive results.^36^ In response to humanitarian crises, trauma-informed and community-based interventions were introduced in Poland, Bosnia and Herzegovina, and Georgia, particularly for war-affected populations.^14^, ^15^, ^16^^,^^37^^,^^38^

Analyses of 17 studies in early detection and intervention demonstrated efforts focused on building primary care capacity,^39^^,^^40^ piloting early intervention services for people at risk of psychosis^41^, ^42^, ^43^, ^44^ and improving access to care for high-risk groups.^45^ Studies from Latvia, Armenia, Croatia, and Czechia showed promising results in enhancing diagnostic skills, supporting structured early interventions, and improving service fidelity.^40^, ^41^, ^42^, ^43^, ^44^ Youth-focused programmes, such as trauma-informed support in Georgia^38^ and web-based interventions for eating disorders in Hungary,^46^ showed early effectiveness in addressing emerging mental health issues.

Finally, very few studies focused on evaluating the impact or of prevention and promotion or early detection and intervention programmes at the national level,^47^, ^48^, ^49^ and only one study reported on policy-making approaches in these areas^50^ (see Appendix for detailed results of the review and references).

### Findings from the expert survey

Mental health prevention, promotion, early detection, and early intervention are recognized as policy priorities across the region. National working groups often support development in this area with varying degrees of engagement from state public health institutions. Common activities include the dissemination of informational materials, provision of online resources, and participation in community-based programmes such as school workshops on addiction, bullying, stress, and healthy lifestyles. For example, Latvia’s Center for Disease Prevention and Control and over 100 Health Promotion Offices in Hungary should implement evidence-based activities. In Lithuania, since 2022, the Ministry of Health has mandated four key programmes—suicide prevention, psychological well-being services, early youth intervention, and addiction counselling—allocating two-thirds of the budget to municipal public health bureaus, which may supplement these programmes based on local indicators and needs.

Some early detection and intervention tasks are assigned to primary care, social services, or schools; however, these sectors are often under-resourced and operate more intuitively than systematically. Nonetheless, countries like Hungary or Ukraine show efforts to integrate mental health components across education, social, and health sectors.

Despite policy-level recognition, implementation remains chronically underfunded at the local level. Non-governmental organizations (NGOs) frequently play a vital role in bridging service gaps. In Poland, organizations led by people with lived experience (PWLE) also contribute. NGOs run anti-stigma campaigns, advocacy initiatives, school and workplace programmes, and self-help groups. However, their sustainability is fragile, as funding largely depends on grants, donations, or self-generated income. For instance, Slovakia’s League for Mental Health receives only about 10% of its funding from the state, limiting opportunities for formal evaluation and long-term planning.

International agencies like the EU, WHO, and UNICEF support many initiatives, but these are usually short-term and project-based, with limited sustainability or scalability. Services often concentrate in large cities and follow foreign models rather than a systemic approach based on local needs assessments and strategic planning. As a result, uncoordinated awareness-raising campaigns—rarely evaluated—remain the most common form of mental health promotion in the region.

#### Crisis services

Crisis hotlines have been developed across the region and they play an important role in mental health protection and early intervention. Yet, these are often categorized as social services and therefore lack funding and multisectoral integration with the mental health care systems across the region. While there are notable exceptions—such as the national crisis hotline currently being launched by the Ministry of Health in Slovakia—most hotlines continue to be operated by non-governmental organizations (NGOs).

Other crisis services such as crisis intervention teams in Slovakia are similarly categorized as social services for various disadvantaged groups. Again, these are most often operated by NGOs and as such they face uncertainties related to funding and sustainability.

#### Education-based programmes

Mental health in schools has gained momentum across the region. School psychologists are present in many countries, with widespread coverage in countries like Azerbaijan. However, their clinical competencies are often limited, and training is not public health oriented therefore services vary in quality, and systematic guidance is rare. In Kosovo, the number of school psychologists is very low, while in Hungary, their relocation to Specialist Pedagogical Services has widened the service gap. In some countries, such as Albania, medical staff in schools also engage in mental health promotion and prevention of risky behaviour alongside emergency care.

A range of mental health and prevention programmes targeting children and youth have been implemented at the national and sub-national levels. In Poland, every school must adopt an Educational-Preventive Program addressing mental health and addiction prevention, supported by a list of recommended initiatives. Croatia’s Life Skills Training program, running for nearly 20 years, promotes students' health and personal development through 40 h of structured content across grades 3–7, reaching about 90% of primary school students in the Primorsko-Goranska County. Despite its scale, no peer-reviewed evaluation is reported. University-based mental health services for students are also common across the region, reflecting a growing recognition of mental health needs in tertiary education settings.

These efforts are rarely complemented by teacher-focused programmes or a comprehensive whole-school approach to mental health. Teacher training typically centres on delivering specific interventions or identifying distress symptoms. External services—such as psychological and pedagogical clinics in Poland or counselling centers in Croatia—often cooperate with schools, but systemic issues persist. These include lack of evaluations, evidence-based guidance, staff training and coordination, unclear roles, insufficient protocols and tools, and limited standardized assessments.

#### Mental health in the workplace

Workplace mental health programmes in the region are still in their early stages and, where implemented, are typically ad hoc—driven by employers' perceived needs or initiated by NGOs rather than embedded within comprehensive strategies. In Slovakia, for instance, the NGO League for Mental Health offers a paid program called the Employers for Mental Health Coalition, which provides materials to help organizations address mental health challenges. In Czechia, some employers have begun investing in employee mental health, though efforts are typically limited to providing access to psychological counselling without broader organizational or policy-level integration.

However, there are also more systematic approaches. For instance, in Poland, excessive stress is required to be addressed by employers and there are programmes, initiatives and materials actively developed and disseminated by state authorities. Family-Friendly Workplace Award in Hungary recognizes companies that support work-life balance and employees’ well-being. Over 500 companies have received this award. Criteria include flexible working arrangements, employee assistance programmes (EAPs), and mental health initiatives. In Romania, workplaces are increasingly recognizing the importance of mental health and taking steps to support their employees’ well-being. Many companies now offer Employee Assistance Programmes (EAPs), giving workers access to counselling and support services to help them cope with stress, mental health challenges, and personal struggles. Workplace workshops and seminars on stress management, mindfulness, and work-life balance are also becoming more common, helping employees build healthier habits. At the policy level, recent legislation is pushing for healthier workplace environments by encouraging equitable workloads, mental health days, and well-being policies.

#### Parenting programmes

While some evidence-based parenting programmes with international origins—such as *Triple P (Positive Parenting Program)* and *The Incredible Years*—have been adopted in several countries, parenting intervention development and uptake remains emergent. A few initiatives, such as the Family Strengthening Program and the School for Parents and Educators in Poland, as well as the previously mentioned programmes in Moldova and North Macedonia, have undergone systematic evaluation. Occasionally, programmes have also been launched with support from WHO, UNICEF, or other international organizations, which assist with funding and implementation. These agencies often play a critical role in providing initial funding, technical guidance, and implementation support. However, the sustainability and long-term integration of such programmes into national systems frequently remain dependent on external assistance, highlighting the need for stronger domestic policy commitment and local leadership and capacity-building in this domain.

#### Primary health care programmes

The essential role of primary health care (PHC) in mental health is increasingly recognized across the region. In countries like Albania, mental health integration into PHC is part of national strategic documents, with concrete steps such as GP training, development of new guidelines, and the creation of model family medicine centres combining health and social care. Albania’s National Check-Up Program targets early detection of depression and alcohol problems in people aged 35–70, though screening is underused.

GPs are often the first point of contact for mental health issues. In Latvia, they are trained to use screening tools like the PHQ-9 for depression, while in Romania, mental health detection and intervention is becoming a routine part of medical visits, with family doctors trained to identify early signs and support mild to moderate conditions. Screening for depression, anxiety, and PTSD is expanding, especially among people having experienced trauma. In Croatia, in addition to care provided by general practitioners, specialized mental health teams offer services for youths and individuals with addiction problems, with centres established in all Croatian counties.

While GPs are authorized to prescribe certain psychotropic medications, such as antidepressants or anxiolytics, insufficient training can lead to problems like overprescription of benzodiazepines. In some countries, like Bulgaria, medications are restricted to psychiatrist prescriptions, which can limit access to timely treatment and create additional barriers within the care pathway.

#### Primary non-health care programmes

Across the region, a broad network of social services and child and youth care institutions—such as residential care homes—has been developed to address a range of social challenges. While these services have traditionally addressed a range of social issues, they are now increasingly encountering clients with mental health difficulties. However, systematic training for social workers in recognizing and managing mental health problems is still rare—Latvia being one of the few exceptions. Despite this, social services remain an essential component of mental health care across the region and, in some cases—such as in Bulgaria—are considered among the best-functioning parts of the mental health system.

There are promising examples of efforts to integrate these services into a broader mental health care system. In Slovakia, for instance, the Central Office for Labor, Social Affairs and Family is implementing a project financed by the European Social Fund aimed at establishing 46 family counselling centres across the country. These centres are intended to operate with multidisciplinary teams and provide psychological support to individuals, couples, and families. However, the project has not yet been fully implemented.

Recognizing the importance of early intervention, Romania has started placing more emphasis on services for children and adolescents. One such initiative is the ‘Open Minds Project,’ which aims to strengthen youth mental health services and engage families and communities in supporting young people’s well-being. However, structured training in basic mental health support for primary care and social service professionals remains rare, limiting system-wide capacity for prevention and early intervention.

#### Perinatal mental health

Perinatal mental health care in the WHO European Region was systematically assessed by Horáková et al.,^51^ ranking countries from 0 to 5 based on the presence of screening, treatment, guidelines, and relevant policies. No CEE country scored above 2, except Poland, which scored a 4. However, significant gaps remain—even in Poland, where mandatory depression screenings during and after pregnancy are often not conducted, as reported by more than two-thirds of mothers.

Despite low scores, some regional initiatives exist. In Hungary, for example, a long-standing health visitor system assigns each pregnant woman a nurse with a specialized healthcare diploma. These nurses provide ongoing monitoring, support, and early screening for maternal mental health issues and childhood developmental disorders. Recent EU-funded programmes have further strengthened their role in perinatal mental health.

#### Early detection and early intervention in psychosis

Early detection and early intervention (EDEI) programmes for psychosis have been piloted and continue to operate in Latvia. In Czechia, they were tested but later discontinued due to setbacks in mental health care reform progress. In Croatia, these services have been operating over a decade as part of psychiatric facilities, typically as separate wards, though they are mostly concentrated in major cities. In most other countries, EDEI is not systematically implemented and remains a marginal activity within other services, such as primary care—falling short of the evidence-based models adopted in many high-income countries.

#### Digital interventions

Across the region, digital mental health resources have been utilized for prevention, promotion and early detection purposes —mainly websites offering information on various mental health issues—are available and typically run by NGOs, though some are managed by state institutions like the National Institute of Mental Health in Czechia. Evidence-based tools, such as iFightDepression, are accessible in countries like Albania, Estonia, Hungary, Ukraine and Kosovo. Estonia also offers anonymous online self-assessments, self-help materials, and e-courses, and is developing a system for real-time mental health monitoring and data sharing. Despite growing interest, the uptake and integration of these tools into routine care remains limited.

#### Suicide prevention

Suicide prevention is among the most developed areas within the broader field of mental health prevention, promotion, early detection, and early intervention. It stands out as the only domain where systematic activities have been implemented across the region. For example, in Georgia, suicide prevention remains the primary focus of mental health-related preventive efforts, while other areas receive significantly less attention. Across the region, suicide prevention initiatives are more likely to be supported by national strategies, specific training programmes, and public awareness campaigns, making them relatively more advanced compared to other areas of mental health care.

## Interpretation

CEE is facing major challenges with profound implications for population mental health. Some of these challenges are regional—such as the war in Ukraine and its consequences—while others reflect global trends, including the digital transformation of society and environmental concerns. These pressures have confronted already fragile mental health care systems, many of which remain burdened by the legacy of their communist past.

Over the past decade, unprecedented efforts have been made across the region to improve mental health care, with new policy documents adopted and, to some extent, implemented. Access to European funding and research initiatives, such as Horizon 2020, has enabled several countries to introduce high-quality services and interventions. Although, such access is largely limited to more affluent countries in the region, this gap has been partially bridged by initiatives supported by international organizations such as WHO and UNICEF. However, in both settings there are problems with sustainability and scalability.

**Fig. 2:**
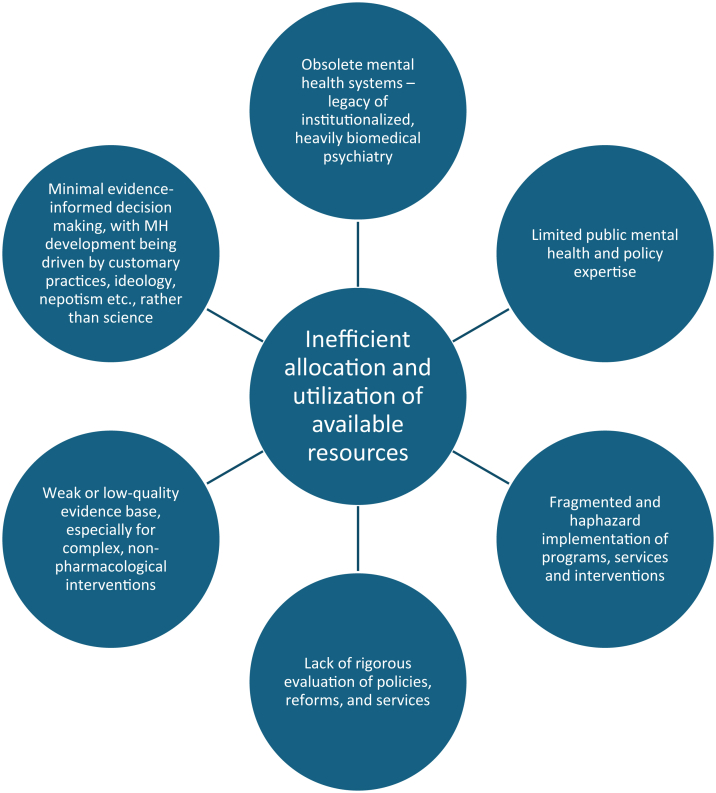
**Reinforcing cycle of suboptimal policy decisions arising from insufficient expertise, limited evidence, and personal biases in Central and Eastern Europe**. This dynamic results in a failure to achieve the maximum attainable level of population mental health within the prevailing structural and resource constraints, as systemic missteps in decision-making (driven by insufficient expertise, evidence gaps, and personal biases) prevent resources from being used in ways that would maximize outcomes.

**Fig. 3:**
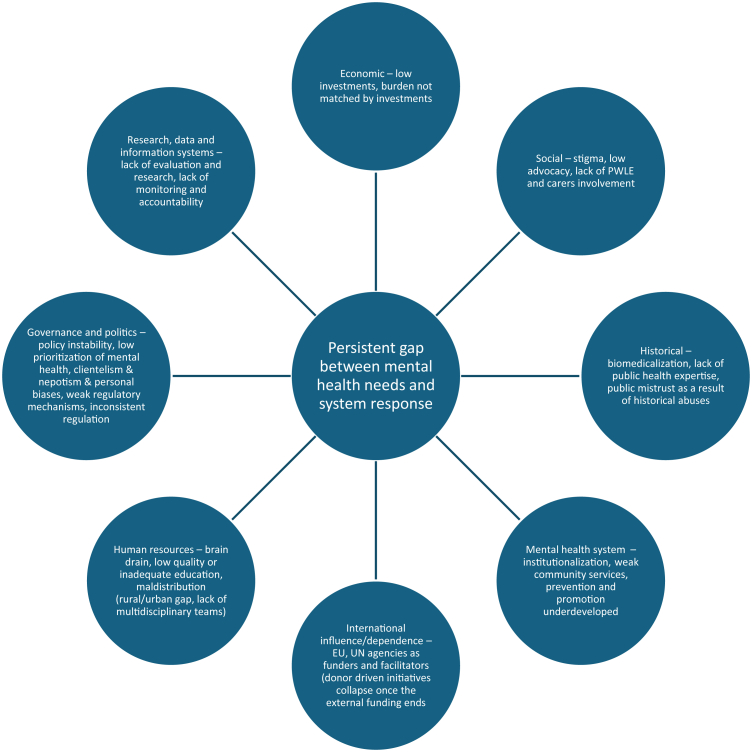
**Structural and contextual barriers to meeting population mental health needs in Central and Eastern Europe**. This results in mental health systems not realizing their full potential in improving population mental health.

In addition, many mental health initiatives and programmes continue to be carried out with a high degree of intuition, improvisation, and limited coordination. A lack of adherence to existing evidence, alongside insufficient generation of new, context-specific research, prevents effective implementation and learning. As a result, significant inefficiencies persist, leading to potential misallocation of scarce resources. In the absence of systematic evaluation and evidence-informed decision-making, opportunities to scale up successful models and phase out ineffective ones are often missed, undermining both impact and sustainability, and contributing to the suboptimal mental health care development (Fig. 2 and Fig. 3).

The war in Ukraine has exposed significant gaps in mental health services across the region. Ukraine’s ongoing efforts to provide MHPSS while simultaneously developing its mental health care system show that, with innovative, adaptable, and evidence-based approaches, systems in CEE can be transformed both rapidly and effectively.^52^

Meanwhile, suicide rates and alcohol consumption remain excessively high in many parts of the region, and the burden of mental health conditions is expected to keep rising. Ukrainian children and adolescents, many living in prolonged exile, might be experiencing growing mental health problems as uncertainty about their future and identity deepens. An unprecedented challenge lies ahead in addressing the mental health needs of Ukrainian military personnel, veterans, their families, and communities. Over one million Ukrainians have joined the Armed Forces, most with frontline experience resulting in severe trauma. Despite efforts from governmental and non-governmental organizations, mental health care remains fragmented and underprepared, with limited recognition of mental health needs among military leadership. Importantly, these challenges will not stay confined to Ukraine. As displaced families reunite across Europe, the psychological consequences of war, including trauma-related mental health issues and increased rates of violence, are likely to spill over into host countries as well.

Strengthening partnerships, sharing experiences, and engaging in collaborative research are essential, and the increased interest in mental health among general populations that is reported across the region^53^ might be a gamechanger —but these efforts must be matched by political will and a genuine commitment to advancing mental health care for all.

## Limitations

Generalizations about CEE, its subregions, or even individual countries should be made with caution, as significant regional disparities—often along rural-urban lines—persist. For example, while Hungary has a nationwide framework for community mental health care, local implementation varies widely, ranging from minimal or absent services in some areas to exemplary integrated bio-psycho-social recovery practices in others.

The reliability of data on the international databases is not always high; for instance, the total population in Kosovo according to the Kosovo Official Registration 2024 is 1.59 million, but the World Bank database states 1.76 million; or the suicide rate for Ukraine is reported at 21.2 per 100,000 population (for 2021) by the World Bank database but only 17.7 by WHO Mental Health Atlas 2020. Furthermore, some data might be derived from inaccurate resource, for instance, the reported number for child psychiatrists in Hungary (9.66 per 100,000 population) appears to be an overestimation, likely based on official registries of licensed professionals rather than active clinical practice. We might not have been able to correct or clarify all the information. MHA 2020 is also approaching being outdated, for instance Albania’s latest mental health action plan pertains to 2023, Ukrainian to 2024; Ukraine now reports mental health to be fully integrated in primary health care via mhGAP approach. Some data are missing from the databases, for instance in Montenegro the rate for child psychiatrists 0.67 per 100,000 population, but this is not reported in MHA. Another limitation is the absence of statistical analyses to explore potential associations between variables such as disease burden and mental health funding; such hypothesis-driven analyses were beyond the scope of this review but warrant future investigation.

## Conclusions

Despite considerable challenges, CEE has advanced mental health systems in recent years, and it has significant opportunities for further improvements. These include extensive networks of social and educational services, the existence of national institutions for health data collection, numerous examples of good practices across various domains of mental health care, relatively high numbers of trained mental health professionals, and increased population interest in mental health. If these assets are effectively leveraged through coordinated, evidence-based strategies, meaningful and rapid progress can be achieved (Panel 1).

Panel 1: Recommendations

• Build on the existing network of social and school services to deliver mental health promotion, prevention, early detection, and early intervention and ensure that these services are integrated; care pathways are clearly defined; and that guidelines are developed and implemented.
• Equip professionals in all health, social and educational services with the necessary mental health knowledge, skills, and competencies.
• To improve sustainability and effectiveness, crisis services should be formally recognized as essential components of mental health systems and funded accordingly.
• Invest in transparent, evidence-based monitoring and evaluation systems to maximize the impact of mental health programmes and initiatives, both existing and future.
• Strengthen integration across health, social, and educational services, fostering a multidisciplinary approach to mental health care.
• Support national institutions working in public health and health information to analyse collected data and generate insights that inform evidence-based mental health care development.
• Promote international exchange of knowledge and experiences and build on best practices from other settings in the region. Too many initiatives still start from scratch, missing the opportunity to apply lessons already learned elsewhere.
• Promote involvement of people with lived experience at all levels of mental health policymaking, implementation, service delivery, monitoring and evaluation.
• Increase the proportion of the healthcare budget allocated to mental health, at minimum to levels comparable with west European and Scandinavian countries, and in line with the share of mental health disorders in the overall burden of disease.

## Contributors

PW led the study and writing. ZG and AK contributed to the design of the study, its conduct and writing. ZG and MP conducted systematic review. AK, PW and LT analyzed country reports. RvV and GT contributed to the study and writing from a perspective of senior experts on mental health in CEE. All other authors created country reports based on an analysis of grey literature and interviews with local experts.

## Use of AI

PW and AK used ChatGPT to improve the grammar, clarity and succinctness of English in this article. After using this tool/service, the authors reviewed and edited the content as needed and take full responsibility for the content of the publication.

## Declaration of interests

Petr Winkler has recently received funding from (a) the European Structural and Investment Funds administered by the Ministry of Labour and Social Affairs and the Ministry of Education, Youth and Sports of the Czech Republic; (b) various Czech ministries; (c) WHO and UNICEF; and (d) the RSJ Foundation. PW has also accepted a new position as Scientific Lead and Team Coordinator of the European Alliance Against Depression. However, this appointment resulted from a competition that concluded after the submission of the present manuscript and has had no influence on its content. Dorottya Őri has received support for attending meetings and/or travel from the Hungarian Psychiatric Association and is a member of this association. She attended the European Congress of Psychiatry in 2024. She holds an unpaid leadership role as a member of the EPA ECPC Research Task Force. She declares no other financial or non-financial conflicts of interest relevant to this work. Róbert Wernigg is employed full-time in a senior management position at the National Directorate-General for Hospitals, Hungary. He has received honoraria and coverage of expenses from Convention Budapest Ltd., Gál Ferenc University, and the Psychoeducation Foundation for lectures and educational events. He has received support (travel, accommodation, registration fees) from the European Psychiatric Association, the Hungarian Psychiatric Association, the WHO, the European Alliance Against Depression, and the EU Joint Action ImpleMENTAL for participation in professional meetings and conferences. He serves as Secretary-General and President elect of the Hungarian Psychiatric Association, is a member of the Supervisory Board of the Hungarian Association of Hygienists, and acts as the WHO National Focal Point of Hungary for Mental Health. He declares no other financial or non-financial conflicts of interest relevant to this work. Karilė Levickaitė, from Lithuania, serves as a Member of the Board and Vice President of Mental Health Europe since May 2024. She declares no other financial or non-financial conflicts of interest relevant to this work. Tomasz M. Gondek has received grants from THCS paid to his institution. He has received payments or honoraria for lectures, presentations, manuscript writing, or educational events from Valeant Polska, Lundbeck Poland, Apotex Poland/Aurovitas Pharma Polska, Celon Pharma, Neuraxpharm Polska, Exeltis Poland, and Takeda Pharma, all paid directly to him. He has also received support for attending meetings and/or travel from Lundbeck Poland, EGIS, and GL Pharma, all paid directly to him. He declares no other financial or non-financial conflicts of interest relevant to this work. Agata Todzia-Kornaś has received grants from THCS paid to her institution. She has received payment or honoraria for lectures, presentations, and manuscript writing, as well as support for attending meetings and/or travel, directly from Aurovitas. She declares no other financial or non-financial conflicts of interest relevant to this work. Graham Thornicroft has recently been supported by the National Institute for Health and Care Research (NIHR) Applied Research Collaboration South London (NIHR ARC South London) at King’s College Hospital NHS Foundation Trust. GT has also recently been supported by the UK Medical Research Council (UKRI) for the Indigo Partnership (MR/R023697/1) awards. The views expressed are those of the authors.
