# Supplementary material for: Mental health in Central and Eastern Europe: a comprehensive analysis
Source: Lancet Reg Health Eur. 2025 Oct 6;57:101464. doi: 10.1016/j.lanepe.2025.101464 (PMC12541642; doi:10.1016/j.lanepe.2025.101464)
Supplement: Appendix [file mmc1.docx]

Supplementary Materials

Contents

[Mental health related DALY 3](#_Toc197422350)

[Methods 4](#_Toc197422351)

[Systematic review details 5](#_Toc197422352)

[Search strategy 5](#_Toc197422353)

[Inclusion and exclusion criteria 7](#_Toc197422354)

[Detailed results 7](#_Toc197422355)

[Data 7](#_Toc197422356)

[MHA analysis 9](#_Toc197422357)

[Mental Health Prevention and Promotion 10](#_Toc197422358)

[Early Detection and Intervention 11](#_Toc197422359)

[Limitations 12](#_Toc197422360)

[References 12](#_Toc197422361)

[Questionnaire: Mental Health and Mental Health Care Systems in Central and Eastern Europe (CEE) 18](#_Toc197422362)

[Introduction 18](#_Toc197422363)

[Methodology 18](#_Toc197422364)

[Section 1: Mental health care systems 22](#_Toc197422365)

[Section 2: Child and adolescent MH 23](#_Toc197422366)

[2.1. Self-Care 23](#_Toc197422367)

[2.2. Informal Community Care 24](#_Toc197422368)

[2.3. Primary Care Mental Health Services 25](#_Toc197422369)

[2.4. Community Mental Health Services 26](#_Toc197422370)

[2.5. Psychiatric services in general hospitals 28](#_Toc197422371)

[2.6. Long-stay facilities and specialist services 29](#_Toc197422372)

[Section 3: Common mental disorders 30](#_Toc197422373)

[3.1. Self-Care 30](#_Toc197422374)

[3.2. Informal Community Care 31](#_Toc197422375)

[3.3. Primary Care Mental Health Services 32](#_Toc197422376)

[3.4. Community Mental Health Services 33](#_Toc197422377)

[3.5. Psychiatric services in general hospitals 35](#_Toc197422378)

[3.6. Long-stay facilities and specialist services 36](#_Toc197422379)

[Section 4: Migrant mental health 37](#_Toc197422380)

[4.1. Self-Care 37](#_Toc197422381)

[4.2. Informal Community Care 38](#_Toc197422382)

[4.3. Primary Care Mental Health Services 39](#_Toc197422383)

[4.4. Community Mental Health Services 40](#_Toc197422384)

[4.5. Psychiatric services in general hospitals 42](#_Toc197422385)

[4.6. Long-stay facilities and specialist services 43](#_Toc197422386)

[Section 5: Mental health promotion and prevention 44](#_Toc197422387)

[Section 6: E-mental health initiatives 46](#_Toc197422388)

# Mental health related DALY

Table 1s DALYs related to mental health problems in CEE as compared to EU14

# Methods

We aimed to map and analyze mental health care in post-communist countries within the WHO Central and Eastern European region (CEE). Excluding Russia, CEE comprises 28 countries, grouped into five subregions covering over 770 million people across 7.5 million km^2^ – Balkans (Albania, Bosnia i Hercegovina (BiH), Bulgaria, Croatia, Kosovo, Montenegro, North Macedonia, Romania, Serbia, and Slovenia), Baltics (Estonia, Latvia, Lithuania), Caucasus (Armenia, Azerbaijan, Georgia), Central Asia (Kazakhstan, Kyrgyzstan, Tajikistan, Turkmenistan, Uzbekistan), Central Europe (Czechia, Hungary, Poland, Slovakia) and Eastern Europe (Belarus, Moldova, Ukraine).

Building on previous reviews (Krupchanka and Winkler, 2018; Aliev et al., 2021), this study focused on filling gaps in the understanding the current state of mental health care systems, namely promotion, prevention, early detection and early intervention. We used two complementary approaches: a dual strategy systematic scoping review and a mixed-methods, multi-country scoping review.

For the systematic review, we followed PRISMA guidelines and conducted two distinct search streams, one on prevention and promotion, the second on early detection and early intervention (see details below). Full search strategies and inclusion/exclusion criteria are in the Appendix. Two reviewers (ZG, MP) independently screened and extracted the data according to a pre-designed table and met iteratively to resolve discrepancies by consensus. Secondary data on system characteristics were drawn from WHO’s Mental Health Atlas (WHO 2017, WHO 2020), the World Bank DataBank (World Bank 2025) and the Global Burden of Disease database (GBD 2025) (all further referred to as ‘databases’).

The multi-country scoping review integrated structured expert-led literature scans with key informant interviews. Collaborators conducted reviews of grey and indexed literature, and conducted interviews with national experts in local languages, capturing cultural and contextual insights. Data collection at the national level followed a standardized reporting questionnaire methodology designed by using the WHO Mental Health Care Pyramid as a structural framework (see Appendix-Questionnaire).

Country reports covered child and adolescent mental health, migrant mental health, and common mental disorders across self-care, informal community care, mental health services in primary and social care, specialized community mental health services, inpatient care in crisis centers and general hospitals, and long-term institutional care. For each level of care, collaborators reported availability of data sources, service provision, funding mechanisms, governance and intersectoral coordination, quality and evaluation mechanisms, gaps and challenges, and sustainability and scalability. Two parallel framework analyses were performed for synthesizing country reports in a regional analysis: one on mental health systems, and one on prevention, promotion, and early intervention.

For system analysis, two reviewers (PW, AK) independently coded reports thematically, generating 29 codes and iteratively developed and refined a codebook. A third reviewer (LT) coded all country reports using binary coding to map each country against the framework across six domains: policy and governance, funding and resources, service delivery and access, workforce and training, social and cultural context, and monitoring, evaluation, and research.

For prevention, promotion, and early intervention, two reviewers (PW, AK) conducted an open coding process using a framework approach. Codes were grouped into eight domains: policy and planning, primary care, schools, self-care, targeted interventions, funding, evaluation, and early detection and intervention. A final codebook of 33 themes was developed and applied by one reviewer (AK) to the reports to extract data on country implementation and initiative across domains mapping the state of implementation and initiatives across domains, ranging from no recognition or activity, to scaled implementation.

Data from both the reviews and global databases were triangulated. Systems findings are reported across each of the six framework domains, while data on prevention, promotion, and early intervention are presented narratively. Discrepancies between data sources were resolved in consultation with country collaborators.

# Systematic review details

## Search strategy

**Databases**

Ovid: PsycINFO, Global Health, Medline, Embase

**Search strategies**

**Prevention and promotion**

Concept: mental health (combine with OR)

- mental health OR mental disorder OR mental illness OR psychological disorders OR psychiatric disorders

Concept: prevention or promotion (combine with OR)

- exp health promotion
- exp primary prevention
- exp secondary prevention
- exp tertiary prevention
- exp prevention

Concept: CEE (combine with OR)

- Central Europe OR Eastern Europe OR Central Eastern Europe OR Albani* OR Bulgaria* OR Belarus* OR Bosnia and Herzegovin* OR Croatia* OR Czechia OR Czech Republic OR Estonia OR Hungar* OR Kosov* OR Latvia* OR Lithuania* OR Macedonia* OR Montenegro* OR Moldov* OR Poland OR Polish OR Romania* OR Slovakia* OR Serbia OR Slovenia* OR Ukrain* OR Armenia* OR Azerbaijan* OR Georgia* OR Kazak* OR Kazakhstan* OR Kyrgyzstan* OR Turkmenistan* OR Uzbek* OR Central Asia

Concept 1 AND Concept 2 AND Concept 3

**Early detection and intervention**

Concept: mental health

- mental health OR mental disorder OR mental illness OR psychological disorders OR psychiatric disorders OR symptoms

Concept: early detection and intervention

- Exp early detection
- Exp early intervention
- Exp screening
- Exp case finding

Concept: CEE

- Central Europe OR Eastern Europe OR Central Eastern Europe OR Albani* OR Bulgaria* OR Belarus* OR Bosnia and Herzegovin* OR Croatia* OR Czechia OR Czech Republic OR Estonia OR Hungar* OR Kosov* OR Latvia* OR Lithuania* OR Macedonia* OR Montenegro* OR Moldov* OR Poland OR Polish OR Romania* OR Slovakia* OR Serbia OR Slovenia* OR Ukrain* OR Armenia* OR Azerbaijan* OR Georgia* OR Kazak* OR Kazakhstan* OR Kyrgyzstan* OR Turkmenistan* OR Uzbek* OR Central Asia

Concept 1 AND Concept 2 AND Concept 3

## Inclusion and exclusion criteria

|  | **Inclusion** | **Exclusion** |
| --- | --- | --- |
| Study design | Systematic reviews, randomized controlled trials, controlled clinical trials, cohort studies, case-control studies, ecological studies, qualitative studies, policy reports. | Case reports, opinion papers |
| Population | Adults, adolescents, children  Countries in CEE | Studies focusing solely on pharmacological interventions without a comprehensive mental health component. |
| Interventions | Mental health promotion, prevention, early detection, and intervention programs targeting perinatal mental health, school, workplace, and digital settings; early intervention services for psychosis; mental health services in primary care and social care; and parenting programs. |  |
| Outcomes | Mental health outcomes (e.g., depression, anxiety, psychosis, suicide), quality of life, service utilization, cost-effectiveness, and implementation factors. |  |

## Detailed results

### Data

We extracted data from databases to describe the burden of mental disorders, alcohol consumption, suicide rates, financing, service infrastructure and workforce capacity.

The dual search strategy yielded 1,993 and 951 references on mental health prevention and promotion, and early detection and intervention respectively. Following de-duplication and full text screening, 50 studies were included: 33 on promotion and prevention, and 17 on early detection and intervention (see Figure 1 for the PRISMA flowchart). The studies on prevention and promotion spanned both epidemiological and implementation research, targeting various populations, including adolescents, parents, healthcare professionals, and the public, across digital and in-person settings.

For the multi-country scoping review, 19 countries reports were collated (Albania, Azerbaijan, Bulgaria, Croatia, Czechia, Estonia, Georgia, Hungary, Kosovo, Kyrgyzstan, Latvia, Lithuania, Moldova, Montenegro, Nort Macedonia, Poland, Romania, Slovakia, and Ukraine) representing all six subregions of CEE.

Figure 1 PRISMA Flowchart - mental health promotion and prevention, and early detection and early intervention in CEE


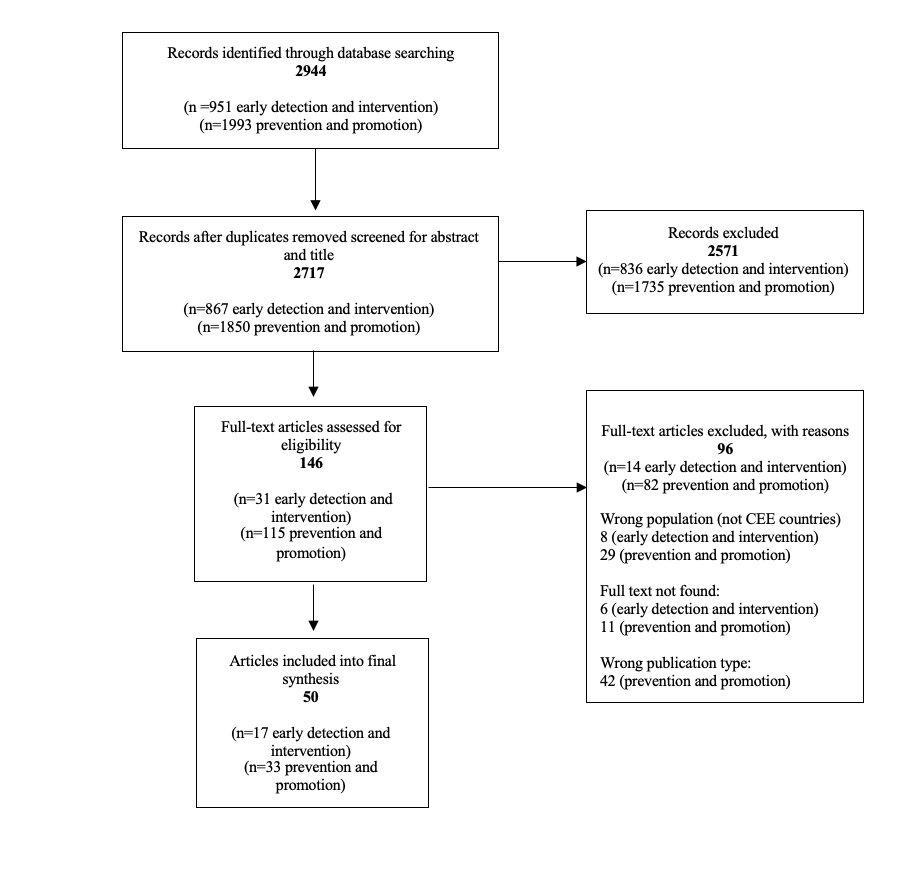


Summary of Characteristics and Key Findings of Included Studies

| Author | Title | Year | Country | Topic | Outcome type | Methods | Participants | Findings |
| --- | --- | --- | --- | --- | --- | --- | --- | --- |
| Giosan et al. | Mental health literacy and academic performance (MHLAP) in high school students: a randomized clinical trial protocol | 2024 | Romania | prevention and promotion | intervention implementation | randomised clinical trial | 264 high school students | Presents the effectiveness of an intervention designed to promote mental health literacy in schools |
| Klein et al. | Risk and protective factors for postpartum depression among Polish women-a prospective study | 2023 | Poland | prevention and promotion | epidemiological study | prospective cohort study | 311 women who gave birth in hospital | Identifies risk and protective factors for PPD among Polish women to help prevent. |
| Roth et al. | A Community-Engaged Approach to Understanding Suicide in a Small Rural County in Georgia: A Two-Phase Content Analysis of Individual and Focus Group Interviews | 2023 | Georgia | prevention and promotion | epidemiological study | qualitative | 22 community stakeholders | Gathers formative research to successfully adapt the most appropriate preventive intervention for suicide to the population and context. |
| Shenderovich et al. | Family-focused intervention to promote adolescent mental health and well-being in Moldova and North Macedonia (FLOURISH): feasibility study protocol | 2023 | Modova, Macedonia | prevention and promotion | intervention implementation | protocol of fesibility study | N/A | Describes adaptation of an intervention, designed to promote adolescent mental health and well-being in Moldova and North Macedonia. |
| Jankowski et al. | One Year On: Poland's Public Health Initiatives and National Response to Millions of Refugees from Ukraine | 2023 | Poland, Ukraine | prevention and promotion | intervention implementation | mixed method | N/A | Examines efugee response in Poland public health inititatives related to Ukrainian refugees |
| Lee et al. | Ukraine refugee crisis: evolving needs and challenges | 2023 | Ukraine | prevention and promotion | intervention implementation | case report | N/A | Examines Ukaine refugee response in Poland |
| Kielan et al. | Promotion of men's mental health | 2020 | Poland | prevention and promotion | intervention implementation | systematic review | N/A | Examines activities for promotion of mens mental health in Poland |
| Franic et al. | Covid-19, child and adolescent mental health - Croatian (in)experience | 2020 | Croatia | prevention and promotion | intervention implementation | review | N/A | Presents perspectives from Croatia regarding Child and Adolescent Mental Health services. |
| Hegerl et al. | Prevention of suicidal behaviour: Results of a controlled community-based intervention study in four European countries | 2019 | Hungary | prevention and promotion | intervention implementation | mixed methods | N/A | Reports on the number of suicides and on results concerning process evaluation analysis |
| Streimann et al. | Effectiveness of a universal classroom-based preventive intervention (PAX GBG): A research protocol for a matched-pair cluster-randomized controlled trial | 2017 | Estonia | prevention and promotion | intervention implementation | cluster-randomized waitlist-controlled trial | 42 schools | Presents research protocol for the study designed to evaluate the impact of a school based intervention on students' mental health and behavior as well as teacher's self-efficacy. |
| Cox et al. | Effectiveness of a trauma/grief-focused group intervention: A qualitative study with war-exposed Bosnian adolescents | 2007 | Bosna and Herzegovina | prevention and promotion | intervention implementation | mixed methods | 66 adolescents | Evaluates the effectiveness of a school-based trauma/grief intervention for adolescents exposed to war, focusing on psychosocial adjustment and skill acquisition. |
| Layne et al. | Trauma/grief-focused group psychotherapy: School-based postwar intervention with traumatized Bosnian adolescents | 2001 | Bosna and Herzegovina | prevention and promotion | intervention implementation | Pre-post test design | 87 secondary school students (ages 15-19) from 17 schools | Evaluatate the effectiveness of trauma/grief-focused group psychotherapy in reducing distress symptoms and promoting positive psychosocial adaptation among war-traumatized adolescents. |
| Obas et al. | Strengthening Primary Healthcare in Kosovo Requires Tailoring Primary, Secondary and Tertiary Prevention Interventions and Consideration of Mental Health. | 2022 | Kosovo | prevention and promotion | intervention implementation | Cross-sectional study design | 977 primary healthcare (PHC) users aged 40 years and above | Assessess primary, secondary, and tertiary prevention indicators for non-communicable diseases (NCDs) in Kosovo and evaluates their association with depressive symptoms. |
| Mihić et al. | Assessing the quality of mental health promotion and prevention in Croatia: the case of Istria | 2017 | Croatia | prevention and promotion | intervention implementation | Cross-sectional study design | N/A | Assessess the quality of mental health promotion and prevention programs in Istria, Croatia, and develops a quality assurance strategy. |
| Lekić et al. | Slovenian practice story: 10 years of e-counselling service for teenagers. | 2011 | Slovenia | prevention and promotion | intervention implementation | Longitudinal approach | 100,000 registered users (adolescents) | Describes an online programme for adolescents in problem-solving and to promote positive mental health, focusing on self-image and social skills. |
| Nordstrom | Ukraine set to act on high suicide burden. | 2007 | Ukraine | prevention and promotion | intervention implementation | review | N/A | Assessess and provides recommendations for improvements of the mental health system in Ukraine, focusing on suicide prevention strategies. |
| Poštuvan et al. | Development and evaluation of online suicide preventive tool iAlive to increase competences in engaging with a suicidal person: Nonrandomized controlled trial. | 2024 | Slovenia | prevention and promotion | intervention implementation | Two-group pre-post test design | 156 participants (50.34%) completed the study | Assesses the effects of the iAlive tool on perceived competences and self-knowledge regarding suicide prevention. |
| Baran et al. | Implementing real-time data suicide surveillance systems. | 2021 | Poland | prevention and promotion | intervention implementation | review | N/A | Advocates for policymakers to support the establishment and implementation of real-time suicide monitoring systems. |
| Streimann et al. | Determines and describes the main features of expertise in the field of mental health in Croatia and its influence on mental health policy-making, ajdnto assess the level of knowledge translation into decision-making and its impact on Croatian mental health policy | 2020 | Estonia | prevention and promotion | intervention implementation | cluster-randomized controlled trial (RCT) | 708 first-grade students from 42 schools | Assesses the impact of school based intervention on students’ mental health and behavior in elementary school and to evaluate whether the intervention leads to improvements in both school and home environments. |
| Novak et al. | Expertise and development of Croatian mental health policy: The perception of mental health professionals. | 2018 | Croatia | prevention and promotion | intervention implementation | Mixed methods design | 121 professionals working in the mental health sector | Determines and describes the main features of expertise in the field of mental health in Croatia and its influences on mental health policy-making, assesses the level of knowledge translation into decision-making and its impact on Croatian mental health policy |
| Patalay et al. | Mental health provision in schools: Approaches and interventions in 10 European countries | 2017 | Poland, Serbia, Ukraine | prevention and promotion | intervention implementation | Cross-sectional study | 1,466 schools | Maps the current approaches to school mental health provision across various European countries, examining specific activities and professionals involved in mental health support in schools. |
| Roškar et al. | Suicide in Slovenia between 1997 and 2010: Characteristics, trends, and preventive activities. | 2015 | Slovenia | prevention and promotion | epidemiological study | longitudinal study | data on 7,317 suicides | Analyzes trends in suicide rates and their characteristics in Slovenia between 1997 and 2010, and examines the effects of preventive activities on the suicide rate in different regions. |
| Surmont et al. | Adolescent suicide and suicide prevention programs: A comparison between Lithuania and Flanders. | 2013 | Lithuania | prevention and promotion | epidemiological study | review | N/A | Compares suicide rates among adolescents in Flanders and Lithuania and discusses trends in public policy initiatives for prevention |
| Milovancevic et al. | The role of mental health professionals contributes to mental health promotion and prevention: Innovative programmes in Serbia. | 2013 | Serbia | prevention and promotion | intervention implementation | review | 750 teachers | Explores the role of mental health professionals in promoting mental health and preventing mental disorders among children and adolescents in Serbia through innovative programs. |
| Roviš | Capacity building for health promotion and drug prevention in Croatia | 2011 | Croatia | prevention and promotion | intervention implementation | planned change model | N/A | Analyzes substance abuse in Croatia and its prevention capacities and practices for dealing with the problem, and explores possibilities for capacity building for health promotion and drug prevention in Croatia. |
| Puras et al. | Child and adolescent mental health in the enlarged European union: Overview of the CAMHEE project. | 2010 | Lithuania, Bulgaria, Romania, Estonia, Latvia, Hungary , Poland, Slovenia | prevention and promotion | intervention implementation | review | N/A | Presents the progress of a European project focused on child and adolescent mental health |
| Fajkic et al. | Child and adolescent suicides in Bosnia and Herzegovina before and after the war (1992-1995). | 2010 | Bosna and Herzegovina | prevention and promotion | epidemiological study | pre-post test design | Prewar period 135 suicides Postwar period 65 suicides | Compares child and adolescent suicides in Bosnia and Herzegovina before and after war in terms of prevalence, sex and age differences, and suicide methods. |
| Hasanović et al. | Post-war mental health promotion in Bosnia-Herzegovina. | 2006 | Bosna and Herzegovina | prevention and promotion | intervention implementation | review | N/A | Describe mental health promotion strategies in Bosnia-Herzegowina post-war. |
| Mishara et al. | Effectiveness of a mental health promotion program to improve coping skills in young children: Zippy's Friends. | 2006 | Lithuania | prevention and promotion | intervention implementation | Quasi-experimental design with pre-test and post-test assessments. | experimental group 314 children from 16 kindergarten classes, control Group 110 children from comparable classes | Evaluates the implementation and effectiveness of the Zippy’s Friends program aimed at improving coping skills and social skills in children. |
| Simovska et al. | Worlds apart or of like minds? Mental health promotion in Macedonian and Australian schools. | 2000 | Macedonia | prevention and promotion | intervention implementation | review | N/A | Descibres mental health promotion in schools in North Macedonia. |
| Lange et al. | Evaluating the Impact of Alcohol Policy on Suicide Mortality: A Sex-Specific Time-Series Analysis for Lithuania | 2023 | Lithuania | prevention and promotion | intervention implementation | generalized additive mixed model | N/A | Tests the impact of three alcohol control policy enactments (in 2008, 2017, and 2018) on suicide mortality among adults aged 25–74 in Lithuania, with a focus on gender differences. |
| Stankunas et al. | Lithuania is tackling health inequalities with support from Norway Grants program | 2017 | Lithuania | prevention and promotion | intervention implementation | Cross-sectional study design | 468 respondents | Develops a model for strengthening capacities to identify and reduce health inequalities in Lithuania. |
| Tucker et al. | Hope Groups: a protocol for a cluster randomized controlled trial of psychosocial, mental health, and parenting support groups for Ukrainian caregivers during war and conflict | 2024 | Ukraine | prevention and promotion | intervention implementation | cluster randomised RCT | Ukrainians externally displaced, internally displaced within Ukraine, and living at home in war‑affected areas | Evaluates the causal effect of 12-session Hope Groups among Ukrainian parents and caregivers affected by war, compared to a waitlist control group |
| Izvoranu et al. | Risk Factors in Postpartum Depression Among Women from South-East of Romania: Importance of Early Diagnosis | 2024 | Romania | early intervention | epidemiological study | observational prospective study | 904 post-partum women | Measures prevalence of post-partum depression |
| Makhashvili et al. | A transdiagnostic psychosocial prevention-intervention service for young people in the Republic of Georgia: early results of the effectiveness study | 2022 | Georgia | early intervention | intervention implementation | quantitative evaluation | 174 young persons, ages 14-25 | Examines the characteristics of young people presenting to Club Synergy an early intervention programme for adolescents and the effectiveness of contact with the service. |
| Mroueh et al. | Can a brief training intervention on schizophrenia and depression improve knowledge, attitudes and practices of primary healthcare workers? The experience in Armenia | 2021 | Armenia | early intervention | intervention implementation | quasi experimental design | !11 GPs and 167 nurses focused on schizophrenia and 459 GPs and 197 nurses focused on depression | Evaluates the effect of a brief intervention on schizophrenia and depression for GPs and nurses |
| Rancans et al. | Prevalence and associated factors of mental disorders in the nationwide primary care population in Latvia: A cross-sectional study | 2020 | Latvia | early intervention | epidemiological study | cross-sectional | 1485 primary care patients | Reports on the current prevalence of mental disorders and suicidality in the nationwide Latvian primary care population |
| Stanetić et al. | Screening of undiagnosed depression among elderly primary care patients: A cross-sectional study from the republic of srpska, Bosnia and Herzegovina | 2020 | Bosna and Herzegovina | early intervention | epidemiological study | cross-sectional | 1198 >65 primary care patients | Determines the prevalence of undiagnosed geriatric depression among primary care patients as well as the sociodemographic and clinical risk factors associated with depression. |
| Šago et al. | Improving the quality of life during treatment in the day hospital for early intervention in Psychiatric Hospital "Sveti Ivan" | 2019 | Croatia | early intervention | intervention implementation | quasi experimental design | 124 patients with psychotic disorders | Examines changes in self-esteem, and quality of life of patients with psychotic disorders treated in a center for early intervention. |
| Srkalović et al. | Prediction of posttraumatic stress disorder symptomatology after childbirth - A Croatian longitudinal study | 2017 | Croatia | early intervention | epidemiological study | observational longitudinal study | 372 post-partum women | Identifies predictors of clinically relevant PTSD symptomatology at 3–5 days and 6–9 weeks following childbirth. |
| Holub et al. | Primary care and the early phases of schizophrenia in the Czech Republic | 2010 | Czechia | early intervention | intervention implementation | cross-sectional | 129 GPs | Explores knowledge, treatment setting, attitudes and needs associated with patients in early phases of psychosis among general practitioners (GPs) in Czech Republic |
| Berze et al. | Implementing the Latvian Early Intervention Program (LAT-EIP) for patients with schizophrenia spectrum first-episode psychosis: Study protocol | 2019 | Latvia | early intervention | intervention implementation | nonrandomized quasi-experimental controlled trial | patients presenting in the psychiatric emergency room with first-time non-affective schizophrenia spectrum psychosis episode | Determines in routine Latvian outpatient settings whether an intervention treatment could be considered as more effective than standard treatment. |
| Gereš et al. | Efficacy of Center for integrative psychiatry multimodal Early Intervention Services in early-phase psychosis on hospital readmission | 2019 | Croatia | early intervention | intervention implementation | retrospective cohort study | 454 patients | Evaluates the effect of the Centre for integrative psychiatry multimodal Early Intervention Services on time to relapse in the patients with early-phase psychosis during 12 and 24 month period. |
| Šago et al. | Day hospital for early intervention for individuals with psychotic disorders | 2018 | Croatia | early intervention | intervention implementation |  |  | complimentary to above describes the setting |
| Matiü et al. | Early intervention services for early-phase psychosis-Centre for integrative psychiatry in psychiatric hospital "Sveti Ivan", Croatia | 2018 | Croatia | early intervention | intervention implementation | retrospective cohort study | 373 early-phase psychosis patients | Evaluates time to relapse of the early-phase psychosis treated in the Center for integrative psychiatry early intervention programs in the period between 2015 and 2017. |
| Restek-Petrović et al. | Psychodynamic group psychoterapy in the early intervention program for patients with psychosis (RIPEPP) | 2018 | Croatia | early intervention | intervention implementation |  |  | complimentary to above describes the setting |
| Restek-Petrović et al. | Early intervention programme for patients with psychotic disorders in "Sveti Ivan". Psychiatric hospital (RIPEPP)-Sociodemographic and baseline characteristics of the participants. | 2017 | Croatia | early intervention | intervention implementation | observational | 245 patients with the first episodes of psychotic disorders, and their family members, participated in the RIPEPP programme. | Describes sociodemographic and baseline characteristics of patients with firs episode psychosis in order to provide better understanding of this population. |
| Szabó et al. | The European initiative ProYouth for the promotion of mental health and the prevention of eating disorders* screening results in Hungary. | 2015 | Hungary | early intervention | intervention implementation | cross-sectional | 801 general population | Explores whether an online programme can address individuals who are at risk for developing eating disorders. |
| Winkler et al. | Value of schizophrenia treatment II: Decision modelling for developing early detection and early intervention services in the Czech Republic. | 2018 | Czechia | early intervention | intervention implementation | decision analytical model | N/A | Shows cost estimates based on an economicmodel for early detection and early interventions services in the Czech Republic. |
| Tomášková et al. | Development and implementation of fidelity assessment in first episode psychosis services in Czechia: A pilot study | 2022 | Czechia | early intervention | intervention implementation | Cross-sectional fidelity assessment | 10 clients and their family members | Assesses the fidelity of early intervention services for first episode psychosis in Czechia and identifies areas for improvement. |

| Author | Title | Year | Country | Topic | Outcome type | Methods | Participants | Findings |
| --- | --- | --- | --- | --- | --- | --- | --- | --- |
| Giosan et al. | Mental health literacy and academic performance (MHLAP) in high school students: a randomized clinical trial protocol | 2024 | Romania | prevention and promotion | intervention implementation | randomised clinical trial | 264 high school students | Presents the effectiveness of an intervention designed to promote mental health literacy in schools |
| Klein et al. | Risk and protective factors for postpartum depression among Polish women-a prospective study | 2023 | Poland | prevention and promotion | epidemiological study | prospective cohort study | 311 women who gave birth in hospital | Identifies risk and protective factors for PPD among Polish women to help prevent. |
| Roth et al. | A Community-Engaged Approach to Understanding Suicide in a Small Rural County in Georgia: A Two-Phase Content Analysis of Individual and Focus Group Interviews | 2023 | Georgia | prevention and promotion | epidemiological study | qualitative | 22 community stakeholders | Gathers formative research to successfully adapt the most appropriate preventive intervention for suicide to the population and context. |
| Shenderovich et al. | Family-focused intervention to promote adolescent mental health and well-being in Moldova and North Macedonia (FLOURISH): feasibility study protocol | 2023 | Modova, Macedonia | prevention and promotion | intervention implementation | protocol of fesibility study | N/A | Describes adaptation of an intervention, designed to promote adolescent mental health and well-being in Moldova and North Macedonia. |
| Jankowski et al. | One Year On: Poland's Public Health Initiatives and National Response to Millions of Refugees from Ukraine | 2023 | Poland, Ukraine | prevention and promotion | intervention implementation | mixed method | N/A | Examines efugee response in Poland public health inititatives related to Ukrainian refugees |
| Lee et al. | Ukraine refugee crisis: evolving needs and challenges | 2023 | Ukraine | prevention and promotion | intervention implementation | case report | N/A | Examines Ukaine refugee response in Poland |
| Kielan et al. | Promotion of men's mental health | 2020 | Poland | prevention and promotion | intervention implementation | systematic review | N/A | Examines activities for promotion of mens mental health in Poland |
| Franic et al. | Covid-19, child and adolescent mental health - Croatian (in)experience | 2020 | Croatia | prevention and promotion | intervention implementation | review | N/A | Presents perspectives from Croatia regarding Child and Adolescent Mental Health services. |
| Hegerl et al. | Prevention of suicidal behaviour: Results of a controlled community-based intervention study in four European countries | 2019 | Hungary | prevention and promotion | intervention implementation | mixed methods | N/A | Reports on the number of suicides and on results concerning process evaluation analysis |
| Streimann et al. | Effectiveness of a universal classroom-based preventive intervention (PAX GBG): A research protocol for a matched-pair cluster-randomized controlled trial | 2017 | Estonia | prevention and promotion | intervention implementation | cluster-randomized waitlist-controlled trial | 42 schools | Presents research protocol for the study designed to evaluate the impact of a school based intervention on students' mental health and behavior as well as teacher's self-efficacy. |
| Cox et al. | Effectiveness of a trauma/grief-focused group intervention: A qualitative study with war-exposed Bosnian adolescents | 2007 | Bosna and Herzegovina | prevention and promotion | intervention implementation | mixed methods | 66 adolescents | Evaluates the effectiveness of a school-based trauma/grief intervention for adolescents exposed to war, focusing on psychosocial adjustment and skill acquisition. |
| Layne et al. | Trauma/grief-focused group psychotherapy: School-based postwar intervention with traumatized Bosnian adolescents | 2001 | Bosna and Herzegovina | prevention and promotion | intervention implementation | Pre-post test design | 87 secondary school students (ages 15-19) from 17 schools | Evaluatate the effectiveness of trauma/grief-focused group psychotherapy in reducing distress symptoms and promoting positive psychosocial adaptation among war-traumatized adolescents. |
| Obas et al. | Strengthening Primary Healthcare in Kosovo Requires Tailoring Primary, Secondary and Tertiary Prevention Interventions and Consideration of Mental Health. | 2022 | Kosovo | prevention and promotion | intervention implementation | Cross-sectional study design | 977 primary healthcare (PHC) users aged 40 years and above | Assessess primary, secondary, and tertiary prevention indicators for non-communicable diseases (NCDs) in Kosovo and evaluates their association with depressive symptoms. |
| Mihić et al. | Assessing the quality of mental health promotion and prevention in Croatia: the case of Istria | 2017 | Croatia | prevention and promotion | intervention implementation | Cross-sectional study design | N/A | Assessess the quality of mental health promotion and prevention programs in Istria, Croatia, and develops a quality assurance strategy. |
| Lekić et al. | Slovenian practice story: 10 years of e-counselling service for teenagers. | 2011 | Slovenia | prevention and promotion | intervention implementation | Longitudinal approach | 100,000 registered users (adolescents) | Describes an online programme for adolescents in problem-solving and to promote positive mental health, focusing on self-image and social skills. |
| Nordstrom | Ukraine set to act on high suicide burden. | 2007 | Ukraine | prevention and promotion | intervention implementation | review | N/A | Assessess and provides recommendations for improvements of the mental health system in Ukraine, focusing on suicide prevention strategies. |
| Poštuvan et al. | Development and evaluation of online suicide preventive tool iAlive to increase competences in engaging with a suicidal person: Nonrandomized controlled trial. | 2024 | Slovenia | prevention and promotion | intervention implementation | Two-group pre-post test design | 156 participants (50.34%) completed the study | Assesses the effects of the iAlive tool on perceived competences and self-knowledge regarding suicide prevention. |
| Baran et al. | Implementing real-time data suicide surveillance systems. | 2021 | Poland | prevention and promotion | intervention implementation | review | N/A | Advocates for policymakers to support the establishment and implementation of real-time suicide monitoring systems. |
| Streimann et al. | Determines and describes the main features of expertise in the field of mental health in Croatia and its influence on mental health policy-making, ajdnto assess the level of knowledge translation into decision-making and its impact on Croatian mental health policy | 2020 | Estonia | prevention and promotion | intervention implementation | cluster-randomized controlled trial (RCT) | 708 first-grade students from 42 schools | Assesses the impact of school based intervention on students’ mental health and behavior in elementary school and to evaluate whether the intervention leads to improvements in both school and home environments. |
| Novak et al. | Expertise and development of Croatian mental health policy: The perception of mental health professionals. | 2018 | Croatia | prevention and promotion | intervention implementation | Mixed methods design | 121 professionals working in the mental health sector | Determines and describes the main features of expertise in the field of mental health in Croatia and its influences on mental health policy-making, assesses the level of knowledge translation into decision-making and its impact on Croatian mental health policy |
| Patalay et al. | Mental health provision in schools: Approaches and interventions in 10 European countries | 2017 | Poland, Serbia, Ukraine | prevention and promotion | intervention implementation | Cross-sectional study | 1,466 schools | Maps the current approaches to school mental health provision across various European countries, examining specific activities and professionals involved in mental health support in schools. |
| Roškar et al. | Suicide in Slovenia between 1997 and 2010: Characteristics, trends, and preventive activities. | 2015 | Slovenia | prevention and promotion | epidemiological study | longitudinal study | data on 7,317 suicides | Analyzes trends in suicide rates and their characteristics in Slovenia between 1997 and 2010, and examines the effects of preventive activities on the suicide rate in different regions. |
| Surmont et al. | Adolescent suicide and suicide prevention programs: A comparison between Lithuania and Flanders. | 2013 | Lithuania | prevention and promotion | epidemiological study | review | N/A | Compares suicide rates among adolescents in Flanders and Lithuania and discusses trends in public policy initiatives for prevention |
| Milovancevic et al. | The role of mental health professionals contributes to mental health promotion and prevention: Innovative programmes in Serbia. | 2013 | Serbia | prevention and promotion | intervention implementation | review | 750 teachers | Explores the role of mental health professionals in promoting mental health and preventing mental disorders among children and adolescents in Serbia through innovative programs. |
| Roviš | Capacity building for health promotion and drug prevention in Croatia | 2011 | Croatia | prevention and promotion | intervention implementation | planned change model | N/A | Analyzes substance abuse in Croatia and its prevention capacities and practices for dealing with the problem, and explores possibilities for capacity building for health promotion and drug prevention in Croatia. |
| Puras et al. | Child and adolescent mental health in the enlarged European union: Overview of the CAMHEE project. | 2010 | Lithuania, Bulgaria, Romania, Estonia, Latvia, Hungary , Poland, Slovenia | prevention and promotion | intervention implementation | review | N/A | Presents the progress of a European project focused on child and adolescent mental health |
| Fajkic et al. | Child and adolescent suicides in Bosnia and Herzegovina before and after the war (1992-1995). | 2010 | Bosna and Herzegovina | prevention and promotion | epidemiological study | pre-post test design | Prewar period 135 suicides Postwar period 65 suicides | Compares child and adolescent suicides in Bosnia and Herzegovina before and after war in terms of prevalence, sex and age differences, and suicide methods. |
| Hasanović et al. | Post-war mental health promotion in Bosnia-Herzegovina. | 2006 | Bosna and Herzegovina | prevention and promotion | intervention implementation | review | N/A | Describe mental health promotion strategies in Bosnia-Herzegowina post-war. |
| Mishara et al. | Effectiveness of a mental health promotion program to improve coping skills in young children: Zippy's Friends. | 2006 | Lithuania | prevention and promotion | intervention implementation | Quasi-experimental design with pre-test and post-test assessments. | experimental group 314 children from 16 kindergarten classes, control Group 110 children from comparable classes | Evaluates the implementation and effectiveness of the Zippy’s Friends program aimed at improving coping skills and social skills in children. |
| Simovska et al. | Worlds apart or of like minds? Mental health promotion in Macedonian and Australian schools. | 2000 | Macedonia | prevention and promotion | intervention implementation | review | N/A | Descibres mental health promotion in schools in North Macedonia. |
| Lange et al. | Evaluating the Impact of Alcohol Policy on Suicide Mortality: A Sex-Specific Time-Series Analysis for Lithuania | 2023 | Lithuania | prevention and promotion | intervention implementation | generalized additive mixed model | N/A | Tests the impact of three alcohol control policy enactments (in 2008, 2017, and 2018) on suicide mortality among adults aged 25–74 in Lithuania, with a focus on gender differences. |
| Stankunas et al. | Lithuania is tackling health inequalities with support from Norway Grants program | 2017 | Lithuania | prevention and promotion | intervention implementation | Cross-sectional study design | 468 respondents | Develops a model for strengthening capacities to identify and reduce health inequalities in Lithuania. |
| Tucker et al. | Hope Groups: a protocol for a cluster randomized controlled trial of psychosocial, mental health, and parenting support groups for Ukrainian caregivers during war and conflict | 2024 | Ukraine | prevention and promotion | intervention implementation | cluster randomised RCT | Ukrainians externally displaced, internally displaced within Ukraine, and living at home in war‑affected areas | Evaluates the causal effect of 12-session Hope Groups among Ukrainian parents and caregivers affected by war, compared to a waitlist control group |
| Izvoranu et al. | Risk Factors in Postpartum Depression Among Women from South-East of Romania: Importance of Early Diagnosis | 2024 | Romania | early intervention | epidemiological study | observational prospective study | 904 post-partum women | Measures prevalence of post-partum depression |
| Makhashvili et al. | A transdiagnostic psychosocial prevention-intervention service for young people in the Republic of Georgia: early results of the effectiveness study | 2022 | Georgia | early intervention | intervention implementation | quantitative evaluation | 174 young persons, ages 14-25 | Examines the characteristics of young people presenting to Club Synergy an early intervention programme for adolescents and the effectiveness of contact with the service. |
| Mroueh et al. | Can a brief training intervention on schizophrenia and depression improve knowledge, attitudes and practices of primary healthcare workers? The experience in Armenia | 2021 | Armenia | early intervention | intervention implementation | quasi experimental design | !11 GPs and 167 nurses focused on schizophrenia and 459 GPs and 197 nurses focused on depression | Evaluates the effect of a brief intervention on schizophrenia and depression for GPs and nurses |
| Rancans et al. | Prevalence and associated factors of mental disorders in the nationwide primary care population in Latvia: A cross-sectional study | 2020 | Latvia | early intervention | epidemiological study | cross-sectional | 1485 primary care patients | Reports on the current prevalence of mental disorders and suicidality in the nationwide Latvian primary care population |
| Stanetić et al. | Screening of undiagnosed depression among elderly primary care patients: A cross-sectional study from the republic of srpska, Bosnia and Herzegovina | 2020 | Bosna and Herzegovina | early intervention | epidemiological study | cross-sectional | 1198 >65 primary care patients | Determines the prevalence of undiagnosed geriatric depression among primary care patients as well as the sociodemographic and clinical risk factors associated with depression. |
| Šago et al. | Improving the quality of life during treatment in the day hospital for early intervention in Psychiatric Hospital "Sveti Ivan" | 2019 | Croatia | early intervention | intervention implementation | quasi experimental design | 124 patients with psychotic disorders | Examines changes in self-esteem, and quality of life of patients with psychotic disorders treated in a center for early intervention. |
| Srkalović et al. | Prediction of posttraumatic stress disorder symptomatology after childbirth - A Croatian longitudinal study | 2017 | Croatia | early intervention | epidemiological study | observational longitudinal study | 372 post-partum women | Identifies predictors of clinically relevant PTSD symptomatology at 3–5 days and 6–9 weeks following childbirth. |
| Holub et al. | Primary care and the early phases of schizophrenia in the Czech Republic | 2010 | Czechia | early intervention | intervention implementation | cross-sectional | 129 GPs | Explores knowledge, treatment setting, attitudes and needs associated with patients in early phases of psychosis among general practitioners (GPs) in Czech Republic |
| Berze et al. | Implementing the Latvian Early Intervention Program (LAT-EIP) for patients with schizophrenia spectrum first-episode psychosis: Study protocol | 2019 | Latvia | early intervention | intervention implementation | nonrandomized quasi-experimental controlled trial | patients presenting in the psychiatric emergency room with first-time non-affective schizophrenia spectrum psychosis episode | Determines in routine Latvian outpatient settings whether an intervention treatment could be considered as more effective than standard treatment. |
| Gereš et al. | Efficacy of Center for integrative psychiatry multimodal Early Intervention Services in early-phase psychosis on hospital readmission | 2019 | Croatia | early intervention | intervention implementation | retrospective cohort study | 454 patients | Evaluates the effect of the Centre for integrative psychiatry multimodal Early Intervention Services on time to relapse in the patients with early-phase psychosis during 12 and 24 month period. |
| Šago et al. | Day hospital for early intervention for individuals with psychotic disorders | 2018 | Croatia | early intervention | intervention implementation |  |  | complimentary to above describes the setting |
| Matiü et al. | Early intervention services for early-phase psychosis-Centre for integrative psychiatry in psychiatric hospital "Sveti Ivan", Croatia | 2018 | Croatia | early intervention | intervention implementation | retrospective cohort study | 373 early-phase psychosis patients | Evaluates time to relapse of the early-phase psychosis treated in the Center for integrative psychiatry early intervention programs in the period between 2015 and 2017. |
| Restek-Petrović et al. | Psychodynamic group psychoterapy in the early intervention program for patients with psychosis (RIPEPP) | 2018 | Croatia | early intervention | intervention implementation |  |  | complimentary to above describes the setting |
| Restek-Petrović et al. | Early intervention programme for patients with psychotic disorders in "Sveti Ivan". Psychiatric hospital (RIPEPP)-Sociodemographic and baseline characteristics of the participants. | 2017 | Croatia | early intervention | intervention implementation | observational | 245 patients with the first episodes of psychotic disorders, and their family members, participated in the RIPEPP programme. | Describes sociodemographic and baseline characteristics of patients with firs episode psychosis in order to provide better understanding of this population. |
| Szabó et al. | The European initiative ProYouth for the promotion of mental health and the prevention of eating disorders* screening results in Hungary. | 2015 | Hungary | early intervention | intervention implementation | cross-sectional | 801 general population | Explores whether an online programme can address individuals who are at risk for developing eating disorders. |
| Winkler et al. | Value of schizophrenia treatment II: Decision modelling for developing early detection and early intervention services in the Czech Republic. | 2018 | Czechia | early intervention | intervention implementation | decision analytical model | N/A | Shows cost estimates based on an economic model for early detection and early interventions services in the Czech Republic. |
| Tomášková et al. | Development and implementation of fidelity assessment in first episode psychosis services in Czechia: A pilot study | 2022 | Czechia | early intervention | intervention implementation | Cross-sectional fidelity assessment | 10 clients and their family members | Assesses the fidelity of early intervention services for first episode psychosis in Czechia and identifies areas for improvement. |

### MHA analysis

According to the Mental Health Atlas 2020, progress in prevention, promotion, and early detection of mental disorders remained limited across the CEE region participating countries. Only a minority of countries reported implementing at least two functioning mental health promotion or prevention programmes; specifically, 18 countries reported having such programmes. This indicates minimal change since the 2017 Atlas, which had similarly low implementation levels. Human resources data revealed persistent gaps in early detection capacity. Though several countries reported the integration of mental health into primary health care, operational coverage, such as community-based facility access, remained inconsistent (WHO 2018; WHO 2021)

### Mental Health Prevention and Promotion

A total of **33 studies** focused on the topic of mental health promotion and prevention, encompassing both **intervention implementation** and **epidemiological studies.** The studies targeted various populations, including adolescents, parents, healthcare professionals, and the general public, using both digital and in-person approaches.

In terms of epidemiological studies, most studies explored **risk and protective factors, social determinants for each countries’ general population or with a focus on specific target groups -i**ncluding adolescents (Lekić et al., 2011; Jankowski et al., 2023; Layne et al., 2001; Cox et al., 2007; Surmont et al., 2013; Milovancevic et al., 2013), parents (Jankowski et al., 2023; Tucker et al., 2024; Shenderovich et al., 2023), men (Kielan et al., 2020), patients with other non-communicable diseases, or comorbidities (Obas et al., 2022; Lange et al., 2023). Perinatal research in Poland identified multiple time-sensitive predictors of postpartum depression (Klein et al. 2024). Another study in Romania (Izvoranu et al., 2024) quantified the prevalence of postpartum depression, underscoring the need for targeted screening and preventive services during pregnancy and early parenthood. Suicide-focused research revealed declining national rates in Slovenia (Roškar et al., 2015), lower-than-expected youth suicides in Bosnia and Herzegovina (Fajkic et al., 2010), and persistent challenges in Lithuania despite existing school-based programmes (Surmont et al., 2013). Qualitative research from Georgia linked suicide risk to rurality, stigma, and limited access to care, emphasising the importance of socio-environmental factors (Roth et al., 2023).

A number of studies focused on school and youth-based prevention programmes (Patalay et al., 2017; Puras et al., 2010; Simovska et al., 2000). In Romania, the SCHOLARS programme (Giosan et al., 2024) was evaluated through a randomized controlled trial involving 264 high school students, with positive outcomes. This web-based intervention aimed to improve mental health literacy and reduce stigma. In Estonia, another randomised controlled trial protocol and subsequent implementation positively evaluated a school based mental health prevention intervention for teachers integrated into the curriculum (Streimann et al., 2017; Streimann et al., 2020). In North Macedonia and Moldova, the Parenting for Lifelong Health programme was culturally adapted for implementation in families with children (Shenderovich et al., 2023), albeit findings are pending. In Lithuania, the programme Zippy’s Friends showed positive outcomes when compared to controls from Denmark (Mishara et al., 2006). Broader health promotion frameworks during COVID-19 (Franic et al., 2020), or related to substance abuse prevention (Roviš, 2011) similarly emphasised the importance of embedding mental health supports in education systems

Several studies reported on community-based and digital health promotion strategies. A decade-long initiative in Slovenia showcased the impact of **online counselling platforms** tailored to adolescents, providing anonymous support and early intervention opportunities via digital communication tools (Lekić et al., 2011). Prevention and promotion initiative were often linked to suicide prevention. A multi-country (including Hungary) community-based suicide prevention programme reported **reductions in suicidal behaviour**, validating large-scale, multicomponent public health efforts (Hegerl et al., 2019). Formative research conducted in Georgia engaged stakeholders to address suicide prevention in rural areas (Roth et al., 2023). Similarly, engagement of policy makers in suicide prevention was reported in Poland (Baran et al., 2021). In Slovenia, an online suicide prevention programme improved participants’ self-perceived competence to respond to suicidal crises (Poštuvan et al., 2024).

Some studies outlined prevention and promotion responses to humanitarian crises such as wars. Evaluations of public health responses in Poland described growing attention to **community-based and trauma-informed approaches following the start of the war in Ukraine**, though detailed results remain unpublished (Jankowski et al., 2023; Lee et al., 2023). Perhaps related to this, one study also reported on suicide prevention for military personnel in Ukraine (this research happened prior to the start of the current war which started in 2022) (Nordstrom, 2007). In Bosnia and Herzegovina, a review showed various post-war mental health promotion activities in the Tuzla canton (Hasanović et al., 2006), a trauma-and grief-focused group programme for war-exposed adolescents led to improved interpersonal relationships and emotional regulation (Cox et al., 2007), and a school-based group psychotherapy study demonstrated significant reductions in posttraumatic stress and grief symptoms (Layne et al., 2001).

Finally, very few studies focused on reporting a larger policy approach to prevention and promotion or evaluation at the national level not at the intervention/programme level. One study from Croatia, presented how key mental health professionals and stakeholders perceive their ability to impact national policymaking, showing a lack of knowledge translation from practice to policy (Novak et al. 2018). Another study focused on evaluating the quality of prevention and promotion activities implemented widely in the Istria region in Croatia, finding critical gaps in evaluation and implementation of local mental health prevention and promotion programmes (Mihić et al., 2017). And another study from Lithuania focused on monitoring and evaluation of health inequalities (including mental health) and programmes related to reduce it (Stankunas et al., 2017).

### **Early Detection and Intervention**

Seventeen studies focused on strategies for early detection and intervention of mental health conditions.

Epidemiological research highlighted **significant diagnostic gaps**. One large-scale Romanian study found that more than **one in five postpartum women** experienced symptoms of depression (Izvoranu et al, 2024), while a Latvian survey found **over 37.2% of primary care patients** screened positive for mental health concerns, yet most lacked access to formal treatment (Rancans et al., 2020). One longitudinal study from Croatia explored the risk factors for post-traumatic stress disorder following childbirth (Imširagić et al., 2017). Among older adults in Bosnia, **40.4% were found to have undiagnosed depression** (Stanetić et al., 2020). A study from Czechia demonstrated that primary care professionals could effectively identify early-stage schizophrenia when supported by structured guidance (Holub et al., 2010).

Most studies describing specific programmes focused on training and capacity-building for early detection. In Armenia, a quasi-experimental study (Mroueh et al., 2021) assessed the impact of a one-day training for general practitioners and nurses on schizophrenia and depression. Results showed improved diagnostic accuracy and knowledge retention, although baseline familiarity with mental illness was low. Interventions for first-episode psychosis in Croatia yielded **positive clinical and psychosocial outcomes (Šago et al., 2018; Šago et al., 2019; Matic et al., 2018)**, also indicating the value of **multimodal early intervention hubs** (Gereš et al., 2019). A more recent Czech national initiative used fidelity assessment tools to monitor community mental health services efforts in early detection and intervetion, achieving moderate fidelity scores and revealing opportunities to strengthen consistency and quality in care delivery (Tomášková et al., 2023).

One study in Latvia piloted a national early intervention programme schizophrenia, with plans to implement a multidisciplinary community-based service to improve access and timely care, though findings were not yet available (Berze et al., 2019). In Croatia, a psychodynamic group therapy programme for individuals in early stages of psychosis showed promise in supporting emotional processing and interpersonal functioning (Restek-Petrović et al., 2017), and a complementary initiative targeting first-episode psychosis patients reported clinical improvement in participants’ mental health and functioning following structured early intervention services (Restek-Petrović et al., 2018).

Youth-focused early intervention programs included an intervention done in Georgia (Club Synergy), which offered trauma-informed psychosocial support to 174 adolescents aged 14–25. The programme, evaluated through a mixed-methods approach, reported promising preliminary outcomes, including reduced trauma symptoms and improved functioning (Makhashvili et al., 2022). In Hungary, a web-based intervention under the ProYouth initiative screened adolescents and young adults for eating disorder risk and severity and provided early intervention (Szabó et al., 2015).

Finally, only one study focused on the evaluation of early detection and early intervention services implementation at the national level (Winkler et al., 2018).

## Limitations

The review has two key limitations, firstly we did not assess the methodological quality or risk of bias of the included studies. Secondly, the literature search was limited to publications in English and internationally indexed sources, excluding local-language databases and regional journals.

## References

Baran A, Gerstner R, Ueda M, Gmitrowicz A. Implementing real-time data suicide surveillance systems. Crisis 2021; published online Sept 16.

Berze L, Civcisa S, Krone I, Kvartalovs D, Kikuste S, Sapele I, et al. Implementing the Latvian Early Intervention Program (LAT-EIP): study protocol. Front Psychiatry 2019; 10: 829.

Cox J, Davies DR, Burlingame GM, Campbell JE, Layne CM, Katzenbach RJ. Effectiveness of a trauma/grief–focused group intervention with war–exposed Bosnian adolescents. Int J Group Psychother 2007; 57: 319–45.

Fajkic A, Lepara O, Voracek M, Kapusta ND, Niederkrotenthaler T, Amiri L, et al. Child and adolescent suicides in Bosnia and Herzegovina before and after the war (1992–1995). Crisis 2010.

Franic T, Dodig-Curkovic K. Covid-19, child and adolescent mental health – Croatian (in)experience. Ir J Psychol Med 2020; 37: 214–17.

Gereš N, Matić K, Prskalo-Čule D, Zadravec Vrbanc T, Lovretić V, Skopljak K, et al. Efficacy of early intervention for psychosis at the Center for Integrative Psychiatry. Psychiatr Danub 2019; 31(Suppl 2): 171–80.

Giosan C, Pană A, Cosmoiu A, Chira AM, Toma AM, Papasteri CC, et al. Mental health literacy and academic performance (MHLAP) in high school students: a randomized clinical trial protocol. Trials 2024; 25: 419.

Hasanović M, Sinanović O, Pajević I, Avdibegović E, Sutović A. Post-war mental health promotion in Bosnia-Herzegovina. Psychiatria Danubina 2006; 18: 74–8.

Hegerl U, Maxwell M, Harris F, Koburger N, Mergl R, Székely A, et al. Prevention of suicidal behaviour: results of a controlled community-based intervention study in four European countries. PLoS One 2019; 14: e0224602.

Holub D, Wenigová B, Umbricht D, Simon AE. Primary care and early-phase schizophrenia in Czechia. Epidemiol Psychiatr Sci 2010; 19: 243–50.

Imširagić AS, Begić D, Šimičević L, Bajić Ž. Prediction of posttraumatic stress disorder symptomatology after childbirth—a Croatian longitudinal study. Women Birth 2017; 30: e17–23.

Izvoranu S, Banariu MG, Chirila S, Nour C, Niculescu C, Rus M, et al. Risk factors in postpartum depression among women from south-east of Romania: importance of early diagnosis. Arch Pharm Pract 2024; 15: 84–90.

Jankowski M, Lazarus JV, Kuchyn I, Zemskov S, Gałązkowski R, Gujski M. One year on: Poland’s public health initiatives and response to Ukrainian refugees. Med Sci Monit 2023; 29: e940223.

Kielan AJ, Stradomska M, Jaworski M, Mosiołek A, Chodkiewicz J, Święcicki Ł, Walewska-Zielecka B. Promotion of men’s mental health. Psychiatria 2020; 17: 212–15.

Klein S, Błażek M, Świetlik D. Risk and protective factors for postpartum depression among Polish women – a prospective study. J Psychosom Obstet Gynaecol 2024; 45: 2291634.

Lange S, Jiang H, Štelemėkas M, Tran A, Cherpitel C, Giesbrecht N, et al. Evaluating the impact of alcohol policy on suicide mortality: a sex-specific time-series analysis for Lithuania. Arch Suicide Res 2023; 27: 339–52.

Layne CM, Pynoos RS, Saltzman WR, Arslanagić B, Black M, Savjak N, et al. Trauma/grief-focused group psychotherapy: school-based postwar intervention in Bosnia. Group Dyn 2001; 5: 277.

Lee AC, Khaw FM, Lindman AE, Juszczyk G. Ukraine refugee crisis: evolving needs and challenges. Public Health 2023; 217: 41–5.

Lekić K, Konec Juričič N, Tratnjek P, Jereb B. Slovenian practice story: 10 years of e-counselling service for teenagers. In: e-Health Across Borders Without Boundaries. IOS Press, 2011: 105–10.

Makhashvili N, Javakhishvili JD, Chikovani I, Bevan-Jones R, Uchaneishvili M, Pilauri K, et al. Transdiagnostic psychosocial prevention-intervention for youth in Georgia: early effectiveness results. Eur J Psychotraumatol 2022; 13: 2060606.

Matic K, Geres N, Gerlach J, Prskalo-Cule D, Zadravec Vrbanc T, Lovretic V. Early intervention services for early-phase psychosis—Centre for integrative psychiatry in Psychiatric Hospital “Sveti Ivan”, Croatia. Psychiatr Danub 2018; 30 (suppl 4): 158–65.

Mihić J, Novak M, Hosman C, Domitrovich C. Assessing the quality of mental health promotion and prevention in Croatia: the case of Istria. Health Promot Int 2017; 32: 511–21.

Milovancevic MP, Jovicic M. The role of mental health professionals contributes to mental health promotion and prevention: innovative programmes in Serbia. *Emotional and Behavioural Difficulties* 2013; 18: 261–9.

Mishara BL, Ystgaard M. Effectiveness of a mental health promotion program to improve coping skills in young children: Zippy’s Friends. Early Child Res Q 2006; 21: 110–23.

Mroueh L, Ekmekdjian D, Aghekyan E, Sukiasyan S, Tadevosyan M, Simonyan V, et al. Brief training on schizophrenia and depression for Armenian primary care workers. Asian J Psychiatry 2021; 66: 102862.

Nordstrom DL. Ukraine set to act on high suicide burden. Inj Prev 2007; 13: 224–6.

Novak M, Petek A. Expertise and development of Croatian mental health policy: the perception of mental health professionals. Socijalna psihijatrija 2018; 46: 343–71.

Obas KA, Bytyci-Katanolli A, Kwiatkowski M, Ramadani Q, Fota N, Jerliu N, et al. Strengthening primary healthcare in Kosovo requires tailoring primary, secondary, and tertiary prevention interventions and consideration of mental health. Front Public Health 2022; 10: 794309.

Patalay P, Gondek D, Moltrecht B, Giese L, Curtin C, Stanković M, et al. Mental health provision in schools: approaches and interventions in 10 European countries. Glob Ment Health 2017; 4: e10.

Poštuvan V, Gomboc V, Čopič Pucihar K, Kljun M, Vičič J, Tančič Grum A, et al. Development and evaluation of online suicide preventive tool iAlive: nonrandomized controlled trial. Crisis 2024; 45: 187–96.

Puras D, Kolaitis G, Tsiantis J. Child and adolescent mental health in the enlarged European Union: overview of the CAMHEE project. Int J Ment Health Promot 2010; 12: 3–9.

Rancans E, Renemane L, Kivite-Urtane A, Ziedonis D. Prevalence and factors of mental disorders in Latvian primary care. Ann Gen Psychiatry 2020; 19: 1–10.

Restek-Petrović B, Majdančić A, Molnar S, Grah M, Ivezić E, Filipčić I, et al. Early intervention for psychosis at Sveti Ivan: baseline participant characteristics. Psychiatr Danub 2017; 29: 162–70.

Restek-Petrović B, Mayer N, Grah M. Psychodynamic group psychotherapy in early psychosis intervention. Psychiatr Danub 2018; 30(Suppl 4): 198–202.

Roškar S, Zorko M, Podlesek A. Suicide in Slovenia between 1997 and 2010. Crisis 2015.

Roth KB, Gaveras E, Ghiathi F, Shaw EK, Shoemaker MS, Howard NA, et al. A community-engaged approach to understanding suicide in a small rural county in Georgia: a two-phase content analysis. Int J Environ Res Public Health 2023; 20: 7145.

Roviš D. Capacity building for health promotion and drug prevention in Croatia. J Public Health 2011; 19: 57–68.

Šago D, Filipčić I, Lovretić V, Mayer N. Day hospital for early intervention for individuals with psychotic disorders. Psychiatria Danubina 2018; 30 (suppl 4): 192–7.

Šago D, Lovretić V, Habuš K, Ivezić E, Bogović Dijaković A, Đogaš VV, Filipčić I. Improving the quality of life during treatment in the Day hospital for early intervention in Psychiatric Hospital "Sveti Ivan". Psychiatria Danubina 2019; 31 (suppl 2): 190–5.

Shenderovich Y, Piolanti A, Babii V, Calovska-Hertzog N, Evans RE, Heinrichs N, et al. Family-focused intervention to promote adolescent mental health and well-being in Moldova and North Macedonia (FLOURISH): feasibility study protocol. BMJ Open 2023; 13: e080400.

Simovska V, Sheehan M. Worlds apart or of like minds? Mental health promotion in Macedonian and Australian schools. Health Educ 2000; 100: 216–23.

Stanetić K, Petrović V, Stanetić B, Kević V, Stanetić M, Matović J, et al. Screening of undiagnosed depression in elderly primary care patients in Bosnia. Med Glas 2020; 17: 200–5.

Stankunas M, Kalediene R. Lithuania is tackling health inequalities with support from Norway Grants program. Public Health 2017; 149: 28–30.

Streimann K, Selart A, Trummal A. Effectiveness of a universal, classroom-based preventive intervention (PAX GBG) in Estonia: a cluster-randomised controlled trial. Prev Sci 2020; 21: 234–44.

Streimann K, Trummal A, Klandorf K, Akkermann K, Sisask M, Toros K, et al. Effectiveness of a universal classroom-based preventive intervention (PAX GBG): a research protocol for a matched-pair cluster-randomised controlled trial. Contemp Clin Trials Commun 2017; 8: 75–84.

Surmont M, Rousseff T, Van Heeringen C, Skokauskas N. Adolescent suicide and suicide prevention programs: a comparison between Lithuania and Flanders. Adolesc Psychiatry 2013; 3

Szabó K, Czeglédi E, Babusa B, Szumska I, Túry F, Sándor I, Bauer S. The ProYouth initiative for mental health promotion and ED prevention: Hungary screening results. Eur Eat Disord Rev 2015; 23: 139–46.

Tomaskova H, Kondrátová L, Winkler P, Addington D. Fidelity assessment in first-episode psychosis services in Czechia: pilot study. Early Interv Psychiatry 2023; 17: 573–80.

Tucker S, Baldonado N, Ruina O, Ratmann O, Flaxman S, Bryn L, et al. Hope Groups: a protocol for a cluster randomised controlled trial of psychosocial, mental health, and parenting support groups for Ukrainian caregivers during war and conflict. *Trials* 2024; 25: 486.

WHO. Mental Health Atlas 2017 [Online]. 2018. Available from: <https://www.who.int/mental_health/evidence/atlas/mental_health_atlas_2017/en/> (accessed April 2024).

WHO. Mental Health Atlas 2020 [Online]. 2021. Available from: <https://www.who.int/publications/i/item/9789240036703> (accessed April 2024).

Winkler P, Broulíková HM, Kondrátová L, Knapp M, Arteel P, Boyer P, et al. Value of schizophrenia treatment II: decision modelling for developing early detection and early intervention services in the Czech Republic. Eur Psychiatry2018; 53: 116–22.

# Questionnaire: Mental Health and Mental Health Care Systems in Central and Eastern Europe (CEE)

## Introduction

This questionnaire aims to gather data on mental health care systems in post-communist countries of the WHO European Region. Our goal is to build upon, rather than repeat, information already available (such as in the Mental Health Atlas or our previous studies). We seek detailed insights that allow for an insider’s perspective on mental health care developments in the region. Specifically, we want to understand what has worked, what has failed, and what hasn’t been addressed at all, to help identify opportunities and future directions.

**Section 1** focuses on mental health care systems. Much of this has been covered in the papers "A blind spot on the global mental health map: a scoping review of 25 years' development of mental health care for people with severe mental illnesses in Central and Eastern Europe"; "Widespread collapse, glimpses of revival: a scoping review of mental health policy and service development in Central Asia", “Mental health plans and policies across the WHO European Region”, and “Implementation of mental health policies and plans across WHO European region: barriers and facilitators” (*the last two papers have not been published yet, but we will share it with you and upload them to the “Literature” folder in Google Drive*). Please review these papers, particularly your country profiles, and build upon what has already been stated, highlighting any notable recent developments.

**Sections 2-4** address specific target groups: children and adolescents, people with common mental disorders, and refugee populations. Again, we do not wish to duplicate data already collected in the WHO Mental Health Atlas. Instead, we aim to understand the "story" behind the data.

**Section 5** focuses on mental health prevention and promotion. While this may seem to overlap with previous sections on specific target groups (sections 2-4), we wanted to ensure nothing is missed. We may reorganize the questionnaire later to avoid repetition.

**Section 6** focuses on e-mental health. As with section 5, it may overlap with previous sections, but again, we are ensuring comprehensive coverage. The questionnaire may be regrouped later to prevent redundancy.

## Methodology

To complete this questionnaire, authors should employ a mixed-methods approach, combining a review of local literature and policy documents with key stakeholder interviews in each country. Co-authors are expected to conduct an in-depth search for relevant national materials, including reports, policy papers, local research articles, and other documents that provide insights into mental health care systems and developments in their respective countries. We aim to gather detailed and up-to-date information that complements existing global data (e.g., the WHO Mental Health Atlas) and previous studies.

In addition to the literature search, co-authors should conduct interviews with relevant stakeholders, such as policymakers, healthcare providers, researchers, representatives of people with lived experience and their families, etc. These interviews should help contextualize the data and provide an insider perspective on the topics below. A minimum of 5 stakeholder interviews is expected for each country to complete the survey, which is designed to cover fields of expertise which stakeholders can be recruited for interview (I.e Section 2 Stakeholder may be a Child Psychiatrist overseeing national child and adolescent mental health services at the MoH). Country co-authors may recruit as many stakeholders as needed to reach data saturation across each section.

It is crucial that co-authors document their process comprehensively, including all sources of information, interviews conducted, and any additional insights gained. This documentation will be used to transparently report our methodology, ensuring that the data collection process is thorough and replicable.

All findings, including literature, data, and interview summaries, should be stored in the appropriate country-specific folder in our shared online environment, ensuring easy access for future reference and analysis.

For sections 2, 3, and 4 of the questionnaire, which focus on child and adolescent mental health, individuals with common mental disorders, and refugees, co-authors are asked to describe the state of the art in their country according to each level of the WHO Mental Health Care Pyramid. This entails providing detailed information for each level separately. Specifically, we request insights on self-care, informal community care, mental health care within primary and social care, specialized community mental health services, inpatient care in crisis centers and general hospitals, and long-term mental health care in facilities.

The following table shows definitions of each level of the WHO Mental Health Care Pyramid, which will be used as a basis for questions in sections 2-4.

Table 2. WHO definitions of levels of WHO Mental Health Care Pyramid

| Self-care | “Most people manage their mental health problems themselves, or with support from family or friends. Self-care is thus the base of the service pyramid, upon which all other care is based. Self-care is most effective when it is supported by formal health services. The formal sector has an essential role in providing information such as how to deal more effectively with stress, the importance of physical activity in staying mentally well, effective ways of dealing with relationships and conflict management, and the dangers of hazardous alcohol and drug use. Self-care should be facilitated through all services and at all levels of the WHO service pyramid.“ (WHO, 2009) |
| --- | --- |
| Informal community care | “Informal community care comprises services provided in the community that are not part of the formal health and welfare system. Examples include traditional healers, professionals in other sectors such as teachers, and police, services provided by nongovernmental organizations, user and family associations, and lay people. This level of care can help prevent relapses among people who have been discharged from hospitals. Informal services are usually accessible and acceptable because they are an integral part of the community. Nonetheless, informal community care should not form the core of mental health service provision, and countries would be ill-advised to depend solely on these services.“ (WHO, 2009) |
| Primary care mental health services | “Mental health care provided within general primary health services is the first level of care within the formal health system. Essential services at this level include early identification and treatment of mental disorders, management of stable psychiatric patients, counselling for common mental disorders, referral to other levels where required, and mental health promotion and prevention activities. Depending on who provides first-level health care in a particular country, general practitioners, nurses or other health workers may provide these assessment, treatment and referral services. Services at the primary health care level are generally the most accessible, affordable and acceptable for communities. Where mental health is integrated as part of these services, access is improved, mental disorders are more likely to be identified and treated, and comorbid physical and mental health problems managed in a seamless way.“ (WHO, 2009) |
| Community mental health services | “Community mental health services include day centres, rehabilitation services, hospital diversion programmes, mobile crisis teams, therapeutic and residential supervised services, group homes, home help, assistance to families, and other support services. Although only some community mental health services will be able to provide the entirety of these services, a combination of some components based on needs and requirements is essential for successful mental health care. In particular, strong community mental health services are essential as part of any deinstitutionalization programme, as well as to prevent unnecessary hospitalization. People receiving good community care have been shown to have better health and mental health outcomes and better quality of life than those treated in psychiatric hospital. To maximize effectiveness, strong links are needed with other services up and down the pyramid of care.“ (WHO, 2009) |
| Psychiatric services in general hospitals | “The development of mental health services in general hospital settings is another essential element of the organization of services. Given the nature of certain mental disorders, hospitalization during acute phases might be required occasionally. District general hospitals provide an accessible and acceptable location for 24-hour medical care and supervision of people with acute worsening of mental disorders, in the same way that these facilities manage acute exacerbations of physical health disorders. Mental health services provided in district general hospitals also enable 24-hour access to services for any physical health problems that might arise during the course of inpatient stays.“ (WHO, 2009) |
| Long-stay facilities and specialist services | “For a small minority of people with mental disorders, specialist care is required beyond that which can be provided in general hospitals. For example, people with treatment-resistant or complex presentations sometimes need to be referred to specialized centres for further testing and treatment. Others occasionally require ongoing care in residential facilities due to their very severe mental disorders or intellectual disabilities and lack of family support. Forensic psychiatry is another type of specialist service that falls into this category. The need for referral to specialist and long-stay services is reduced when general hospitals are staffed with highly-specialized health workers such as psychiatrists and psychologists. However, this is seldom possible in low-income countries, where ratios of mental health professionals to the population are very low. Long stay and specialist service facilities should not be equated with the psychiatric hospitals that dominated mental health care through most of the 20th century. Psychiatric hospitals have a history of serious human rights violations, poor clinical outcomes, and inadequate rehabilitation programmes. They also are costly and consume a disproportionate proportion of mental health expenditures. WHO recommends that psychiatric hospitals be closed and replaced by services in general hospitals, community mental health services, and services integrated into primary health care.“ (WHO, 2009) |

For each of these domains, we are interested in the services available, their sources of funding, governance, accessibility and availability, and how they are regulated. Additionally, we ask for any epidemiological data, promising practices or studies conducted in that sector, as well as any barriers or challenges faced.

## Section 1: Mental health care systems

- **How would you describe the current state of the mental health care system in your country?**
  - **Key Data:** Provide any relevant studies, reports, or statistics.
  - **Planning and decision making**: What is the process for planning of mental health services? Who are responsible and drivers (i.e local and international organizations and influences). What factors impact the decision-making process for service planning (i.e. politics, evidence-based decision making protocols, public interest, availability of funding)? Is the planning consistent and transparent or ad-hoc?
  - **Funding:** How is the mental health system funded (e.g., government, private, donor-funded)?
  - **Governance and cooperation:** Who governs the mental health system in your country? How are these services governed? Who are the key agencies/organizations comprising the network of services, and how do they collaborate with one another?
  - **Quality and Evaluation:** is the performance of the mental health system assessed regularly? Are the data being taken into account when planning and decision.making?
  - **Gaps or Challenges:** Highlight any major gaps, barriers, or challenges faced in the structuring of mental health systems.
- **What are recent events, disruptions, or influences impacting mental health care system transformations in your country? What changes have they led to?**

Briefly describe any events (e.g., natural disasters, war, major societal changes, political unrest or instability, COVID-19) that have impacted population mental health and the mental health care system in your country (200-300 words).

In your response, please address the following:

- The nature of the event
- Its impact on population mental health and the mental health care system
- Any notable responses or interventions that have emerged as a result
- **What major mental health care reforms have been initiated in your country in the past decade?**
  - Description of reforms
  - Goals of the reforms
  - Outcomes or impacts observed so far
- Prompts: Deinstitutionalization; inclusion of people with lived experience (PWLE); financing; service provision and development; human rights; data and research
- **Have there been any other notable recent developments in mental health care policies or practices since 2017?**
  - Yes/No
  - If yes, please describe (200-300 words)
- **What are the current priorities for mental health care reform in your country?**
  - Key areas of focus
  - Challenges in implementation

## Section 2: Child and adolescent MH

Please describe the state of child and adolescent mental health in your country at each level of the WHO Mental Health Care Pyramid.

Target group: All populations <19.

### 2.1. Self-Care

Describe all activities which fall under the WHO definition of self-care below ([definition](#bookmark=id.pfbfx048q17p)).

In your description, include:

- **General Overview:** What services or programs exist?

- **Key Data:** Provide any relevant studies, reports, or statistics.

- **Planning and decision making**: What is the process for planning of mental health services? What factors impact the decision-making process for service planning (i.e. politics, evidence-based decision making protocols, public interest, sustainability, availability of funding)? Is the planning consistent and transparent or ad-hoc?

- **Availability:** Are these services widely accessible or limited to certain areas?

- **Funding:** How are these services funded (e.g., government, private, donor-funded)?

- **Governance and cooperation:** How are these services governed? How are they integrated into the existing network of services, and how do they collaborate with one another?

- **Quality and Evaluation:** What is known about the quality of these services? Are there formal evaluations? (Are services monitored and evaluated for: feasibility and acceptability, need and accessibility, service-user evaluations, fidelity and impact evaluations, cost-effectiveness)

- **Gaps or Challenges:** Highlight any major gaps, barriers, or challenges faced in this area.

- **Sustainability and scalability**: Are the services sustainable in the long-term (consider trends political will, financing, human resources, coverage, access)? What are key barriers in scalability (substantial increase of availability and coverage services) in your country?

**Description (up to 500 words):**

**SWOT:**

a) Strengths

b) Weaknesses

c) Opportunities

d) Threats

### 2.2. Informal Community Care

Describe all activities which fall under the WHO definition of self-care below ([definition](#bookmark=id.lp0tu6x46zbm)).

In your description, include:

- **General Overview:** What services or programs exist?

- **Key Data:** Provide any relevant studies, reports, or statistics.

- **Planning and decision making**: What is the process for planning of mental health services? What factors impact the decision-making process for service planning (i.e. politics, evidence-based decision making protocols, public interest, availability of funding)? Is the planning consistent and transparent or ad-hoc?

- **Availability:** Are these services widely accessible or limited to certain areas?

- **Funding:** How are these services funded (e.g., government, private, donor-funded)?

- **Governance and cooperation:** How are these services governed? How are they integrated into the existing network of services, and how do they collaborate with one another?

- **Quality and Evaluation:** What is known about the quality of these services? Are there formal evaluations? (Are services monitored and evaluated for: feasibility and acceptability, need and accessibility, service-user evaluations, fidelity and impact evaluations, cost-effectiveness)

- **Gaps or Challenges:** Highlight any major gaps, barriers, or challenges faced in this area.

- **Sustainability and scalability**: Are the services sustainable in the long-term (consider trends political will, financing, human resources, coverage, access)? What are key barriers in scalability (substantial increase of availability and coverage services) in your country?

**Description (up to 500 words):**

**SWOT:**

a) Strengths

b) Weaknesses

c) Opportunities

d) Threats

### 2.3. Primary Care Mental Health Services

Describe all activities which fall under the WHO definition of primary care mental health services below ([definition](#bookmark=id.m30z10bruzte)).

In your description, include:

- **General Overview:** What services or programs exist?

- **Key Data:** Provide any relevant studies, reports, or statistics.

- **Planning and decision making**: What is the process for planning of mental health services? What factors impact the decision-making process for service planning (i.e. politics, evidence-based decision making protocols, public interest, availability of funding)? Is the planning consistent and transparent or ad-hoc?

- **Availability:** Are these services widely accessible or limited to certain areas?

- **Funding:** How are these services funded (e.g., government, private, donor-funded)?

- **Governance and cooperation:** How are these services governed? How are they integrated into the existing network of services, and how do they collaborate with one another?

- **Quality and Evaluation:** What is known about the quality of these services? Are there formal evaluations? (Are services monitored and evaluated for: feasibility and acceptability, need and accessibility, service-user evaluations, fidelity and impact evaluations, cost-effectiveness)

- **Gaps or Challenges:** Highlight any major gaps, barriers, or challenges faced in this area.

- **Sustainability and scalability**: Are the services sustainable in the long-term (consider trends political will, financing, human resources, coverage, access)? What are key barriers in scalability (substantial increase of availability and coverage services) in your country?

**Description (up to 500 words)**

**SWOT:**

a) Strengths

b) Weaknesses

c) Opportunities

d) Threats

### 2.4. Community Mental Health Services

Describe all activities which fall under the WHO definition of crisis services below ([definition](#bookmark=id.n5ocobonfw12)).

**a) Crisis Services**

- **General Overview:** What services or programs exist?

- **Key Data:** Provide any relevant studies, reports, or statistics.

- **Planning and decision making**: What is the process for planning of mental health services? What factors impact the decision-making process for service planning (i.e. politics, evidence-based decision making protocols, public interest, availability of funding)? Is the planning consistent and transparent or ad-hoc?

- **Availability:** Are these services widely accessible or limited to certain areas?

- **Funding:** How are these services funded (e.g., government, private, donor-funded)?

- **Governance and cooperation:** How are these services governed? How are they integrated into the existing network of services, and how do they collaborate with one another?

- **Quality and Evaluation:** What is known about the quality of these services? Are there formal evaluations? (Are services monitored and evaluated for: feasibility and acceptability, need and accessibility, service-user evaluations, fidelity and impact evaluations, cost-effectiveness)

- **Gaps or Challenges:** Highlight any major gaps, barriers, or challenges faced in this area.

- **Sustainability and scalability**: Are the services sustainable in the long-term (consider trends political will, financing, human resources, coverage, access)? What are key barriers in scalability (substantial increase of availability and coverage services) in your country?

**Description (up to 500 words)**

**SWOT:**

a) Strengths

b) Weaknesses

c) Opportunities

d) Threats

**b) Specialized Community Mental Health Services**

- **General Overview:** What services or programs exist?

- **Key Data:** Provide any relevant studies, reports, or statistics.

- **Planning and decision making**: What is the process for planning of mental health services? What factors impact the decision-making process for service planning (i.e. politics, evidence-based decision making protocols, public interest, availability of funding)? Is the planning consistent and transparent or ad-hoc?

- **Availability:** Are these services widely accessible or limited to certain areas?

- **Funding:** How are these services funded (e.g., government, private, donor-funded)?

- **Governance and cooperation:** How are these services governed? How are they integrated into the existing network of services, and how do they collaborate with one another?

- **Quality and Evaluation:** What is known about the quality of these services? Are there formal evaluations? (Are services monitored and evaluated for: feasibility and acceptability, need and accessibility, service-user evaluations, fidelity and impact evaluations, cost-effectiveness)

- **Gaps or Challenges:** Highlight any major gaps, barriers, or challenges faced in this area.

- **Sustainability and scalability**: Are the services sustainable in the long-term (consider trends political will, financing, human resources, coverage, access)? What are key barriers in scalability (substantial increase of availability and coverage services) in your country?

**Description (up to 500 words):**

**SWOT:**

a) Strengths

b) Weaknesses

c) Opportunities

d) Threats

### 2.5. Psychiatric services in general hospitals

Describe all activities which fall under the WHO definition of psychiatric services in general hospitals below ([definition](#bookmark=id.cjr4q97qz8s5)).

In your description, include:

- **General Overview:** What services or programs exist?

- **Key Data:** Provide any relevant studies, reports, or statistics.

- **Planning and decision making**: What is the process for planning of mental health services? What factors impact the decision-making process for service planning (i.e. politics, evidence-based decision making protocols, public interest, availability of funding)? Is the planning consistent and transparent or ad-hoc?

- **Availability:** Are these services widely accessible or limited to certain areas?

- **Funding:** How are these services funded (e.g., government, private, donor-funded)?

- **Governance and cooperation:** How are these services governed? How are they integrated into the existing network of services, and how do they collaborate with one another?

- **Quality and Evaluation:** What is known about the quality of these services? Are there formal evaluations? (Are services monitored and evaluated for: feasibility and acceptability, need and accessibility, service-user evaluations, fidelity and impact evaluations, cost-effectiveness)

- **Gaps or Challenges:** Highlight any major gaps, barriers, or challenges faced in this area.

- **Sustainability and scalability**: Are the services sustainable in the long-term (consider trends political will, financing, human resources, coverage, access)? What are key barriers in scalability (substantial increase of availability and coverage services) in your country?

**Description (up to 500 words):**

**SWOT:**

a) Strengths

b) Weaknesses

c) Opportunities

d) Threats

### 2.6. Long-stay facilities and specialist services

Describe all activities which fall under the WHO definition of long-stay facilities and specialist services in general hospitals below ([definition](#bookmark=id.ri0ijgp2xnlc)).

In your description, include:

- **General Overview:** What services or programs exist?

- **Key Data:** Provide any relevant studies, reports, or statistics.

- **Planning and decision making**: What is the process for planning of mental health services? What factors impact the decision-making process for service planning (i.e. politics, evidence-based decision making protocols, public interest, availability of funding)? Is the planning consistent and transparent or ad-hoc?

- **Availability:** Are these services widely accessible or limited to certain areas?

- **Funding:** How are these services funded (e.g., government, private, donor-funded)?

- **Governance and cooperation:** How are these services governed? How are they integrated into the existing network of services, and how do they collaborate with one another?

- **Quality and Evaluation:** What is known about the quality of these services? Are there formal evaluations? (Are services monitored and evaluated for: feasibility and acceptability, need and accessibility, service-user evaluations, fidelity and impact evaluations, cost-effectiveness)

- **Gaps or Challenges:** Highlight any major gaps, barriers, or challenges faced in this area.

- **Sustainability and scalability**: Are the services sustainable in the long-term (consider trends political will, financing, human resources, coverage, access)? What are key barriers in scalability (substantial increase of availability and coverage services) in your country?

**Description (up to 500 words):**

**SWOT:**

a) Strengths

b) Weaknesses

c) Opportunities

d) Threats

## Section 3: Common mental disorders

Please describe the state of common mental disorders services in your country at each level of the WHO Mental Health Care Pyramid.

Target group: Populations with depression, anxiety.

### 3.1. Self-Care

Describe all activities which fall under the WHO definition of self-care below ([definition](#bookmark=id.pfbfx048q17p)).

In your description, include:

- **General Overview:** What services or programs exist?

- **Key Data:** Provide any relevant studies, reports, or statistics.

- **Planning and decision making**: What is the process for planning of mental health services? What factors impact the decision-making process for service planning (i.e. politics, evidence-based decision making protocols, public interest, sustainability, availability of funding)? Is the planning consistent and transparent or ad-hoc?

- **Availability:** Are these services widely accessible or limited to certain areas?

- **Funding:** How are these services funded (e.g., government, private, donor-funded)?

- **Governance and cooperation:** How are these services governed? How are they integrated into the existing network of services, and how do they collaborate with one another?

- **Quality and Evaluation:** What is known about the quality of these services? Are there formal evaluations? (Are services monitored and evaluated for: feasibility and acceptability, need and accessibility, service-user evaluations, fidelity and impact evaluations, cost-effectiveness)

- **Gaps or Challenges:** Highlight any major gaps, barriers, or challenges faced in this area.

- **Sustainability and scalability**: Are the services sustainable in the long-term (consider trends political will, financing, human resources, coverage, access)? What are key barriers in scalability (substantial increase of availability and coverage services) in your country?

**Description (up to 500 words):**

**SWOT:**

a) Strengths

b) Weaknesses

c) Opportunities

d) Threats

### 3.2. Informal Community Care

Describe all activities which fall under the WHO definition of self-care below ([definition](#bookmark=id.lp0tu6x46zbm)).

In your description, include:

- **General Overview:** What services or programs exist?

- **Key Data:** Provide any relevant studies, reports, or statistics.

- **Planning and decision making**: What is the process for planning of mental health services? What factors impact the decision-making process for service planning (i.e. politics, evidence-based decision making protocols, public interest, availability of funding)? Is the planning consistent and transparent or ad-hoc?

- **Availability:** Are these services widely accessible or limited to certain areas?

- **Funding:** How are these services funded (e.g., government, private, donor-funded)?

- **Governance and cooperation:** How are these services governed? How are they integrated into the existing network of services, and how do they collaborate with one another?

- **Quality and Evaluation:** What is known about the quality of these services? Are there formal evaluations? (Are services monitored and evaluated for: feasibility and acceptability, need and accessibility, service-user evaluations, fidelity and impact evaluations, cost-effectiveness)

- **Gaps or Challenges:** Highlight any major gaps, barriers, or challenges faced in this area.

- **Sustainability and scalability**: Are the services sustainable in the long-term (consider trends political will, financing, human resources, coverage, access)? What are key barriers in scalability (substantial increase of availability and coverage services) in your country?

**Description (up to 500 words):**

**SWOT:**

a) Strengths

b) Weaknesses

c) Opportunities

d) Threats

### 3.3. Primary Care Mental Health Services

Describe all activities which fall under the WHO definition of primary care mental health services below ([definition](#bookmark=id.m30z10bruzte)).

In your description, include:

- **General Overview:** What services or programs exist?

- **Key Data:** Provide any relevant studies, reports, or statistics.

- **Planning and decision making**: What is the process for planning of mental health services? What factors impact the decision-making process for service planning (i.e. politics, evidence-based decision making protocols, public interest, availability of funding)? Is the planning consistent and transparent or ad-hoc?

- **Availability:** Are these services widely accessible or limited to certain areas?

- **Funding:** How are these services funded (e.g., government, private, donor-funded)?

- **Governance and cooperation:** How are these services governed? How are they integrated into the existing network of services, and how do they collaborate with one another?

- **Quality and Evaluation:** What is known about the quality of these services? Are there formal evaluations? (Are services monitored and evaluated for: feasibility and acceptability, need and accessibility, service-user evaluations, fidelity and impact evaluations, cost-effectiveness)

- **Gaps or Challenges:** Highlight any major gaps, barriers, or challenges faced in this area.

- **Sustainability and scalability**: Are the services sustainable in the long-term (consider trends political will, financing, human resources, coverage, access)? What are key barriers in scalability (substantial increase of availability and coverage services) in your country?

**Description (up to 500 words)**

**SWOT:**

a) Strengths

b) Weaknesses

c) Opportunities

d) Threats

### 3.4. Community Mental Health Services

Describe all activities which fall under the WHO definition of crisis services below ([definition](#bookmark=id.n5ocobonfw12)).

**a) Crisis Services**

- **General Overview:** What services or programs exist?

- **Key Data:** Provide any relevant studies, reports, or statistics.

- **Planning and decision making**: What is the process for planning of mental health services? What factors impact the decision-making process for service planning (i.e. politics, evidence-based decision making protocols, public interest, availability of funding)? Is the planning consistent and transparent or ad-hoc?

- **Availability:** Are these services widely accessible or limited to certain areas?

- **Funding:** How are these services funded (e.g., government, private, donor-funded)?

- **Governance and cooperation:** How are these services governed? How are they integrated into the existing network of services, and how do they collaborate with one another?

- **Quality and Evaluation:** What is known about the quality of these services? Are there formal evaluations? (Are services monitored and evaluated for: feasibility and acceptability, need and accessibility, service-user evaluations, fidelity and impact evaluations, cost-effectiveness)

- **Gaps or Challenges:** Highlight any major gaps, barriers, or challenges faced in this area.

- **Sustainability and scalability**: Are the services sustainable in the long-term (consider trends political will, financing, human resources, coverage, access)? What are key barriers in scalability (substantial increase of availability and coverage services) in your country?

**Description (up to 500 words)**

**SWOT:**

a) Strengths

b) Weaknesses

c) Opportunities

d) Threats

**b) Specialized Community Mental Health Services**

- **General Overview:** What services or programs exist?

- **Key Data:** Provide any relevant studies, reports, or statistics.

- **Planning and decision making**: What is the process for planning of mental health services? What factors impact the decision-making process for service planning (i.e. politics, evidence-based decision making protocols, public interest, availability of funding)? Is the planning consistent and transparent or ad-hoc?

- **Availability:** Are these services widely accessible or limited to certain areas?

- **Funding:** How are these services funded (e.g., government, private, donor-funded)?

- **Governance and cooperation:** How are these services governed? How are they integrated into the existing network of services, and how do they collaborate with one another?

- **Quality and Evaluation:** What is known about the quality of these services? Are there formal evaluations? (Are services monitored and evaluated for: feasibility and acceptability, need and accessibility, service-user evaluations, fidelity and impact evaluations, cost-effectiveness)

- **Gaps or Challenges:** Highlight any major gaps, barriers, or challenges faced in this area.

- **Sustainability and scalability**: Are the services sustainable in the long-term (consider trends political will, financing, human resources, coverage, access)? What are key barriers in scalability (substantial increase of availability and coverage services) in your country?

**Description (up to 500 words):**

**SWOT:**

a) Strengths

b) Weaknesses

c) Opportunities

d) Threats

### 3.5. Psychiatric services in general hospitals

Describe all activities which fall under the WHO definition of psychiatric services in general hospitals below ([definition](#bookmark=id.cjr4q97qz8s5)).

In your description, include:

- **General Overview:** What services or programs exist?

- **Key Data:** Provide any relevant studies, reports, or statistics.

- **Planning and decision making**: What is the process for planning of mental health services? What factors impact the decision-making process for service planning (i.e. politics, evidence-based decision making protocols, public interest, availability of funding)? Is the planning consistent and transparent or ad-hoc?

- **Availability:** Are these services widely accessible or limited to certain areas?

- **Funding:** How are these services funded (e.g., government, private, donor-funded)?

- **Governance and cooperation:** How are these services governed? How are they integrated into the existing network of services, and how do they collaborate with one another?

- **Quality and Evaluation:** What is known about the quality of these services? Are there formal evaluations? (Are services monitored and evaluated for: feasibility and acceptability, need and accessibility, service-user evaluations, fidelity and impact evaluations, cost-effectiveness)

- **Gaps or Challenges:** Highlight any major gaps, barriers, or challenges faced in this area.

- **Sustainability and scalability**: Are the services sustainable in the long-term (consider trends political will, financing, human resources, coverage, access)? What are key barriers in scalability (substantial increase of availability and coverage services) in your country?

**Description (up to 500 words):**

**SWOT:**

a) Strengths

b) Weaknesses

c) Opportunities

d) Threats

### 3.6. Long-stay facilities and specialist services

Describe all activities which fall under the WHO definition of long-stay facilities and specialist services in general hospitals below ([definition](#bookmark=id.ri0ijgp2xnlc)).

In your description, include:

- **General Overview:** What services or programs exist?

- **Key Data:** Provide any relevant studies, reports, or statistics.

- **Planning and decision making**: What is the process for planning of mental health services? What factors impact the decision-making process for service planning (i.e. politics, evidence-based decision making protocols, public interest, availability of funding)? Is the planning consistent and transparent or ad-hoc?

- **Availability:** Are these services widely accessible or limited to certain areas?

- **Funding:** How are these services funded (e.g., government, private, donor-funded)?

- **Governance and cooperation:** How are these services governed? How are they integrated into the existing network of services, and how do they collaborate with one another?

- **Quality and Evaluation:** What is known about the quality of these services? Are there formal evaluations? (Are services monitored and evaluated for: feasibility and acceptability, need and accessibility, service-user evaluations, fidelity and impact evaluations, cost-effectiveness)

- **Gaps or Challenges:** Highlight any major gaps, barriers, or challenges faced in this area.

- **Sustainability and scalability**: Are the services sustainable in the long-term (consider trends political will, financing, human resources, coverage, access)? What are key barriers in scalability (substantial increase of availability and coverage services) in your country?

**Description (up to 500 words):**

**SWOT:**

a) Strengths

b) Weaknesses

c) Opportunities

d) Threats

## Section 4: Migrant mental health

Please describe the state of migrant mental health services in your country at each level of the WHO Mental Health Care Pyramid.

Target group: Populations include internally displaced people, refugees, immigrants, emigrants

### 4.1. Self-Care

Describe all activities which fall under the WHO definition of self-care below ([definition](#bookmark=id.pfbfx048q17p)).

In your description, include:

- **General Overview:** What services or programs exist?

- **Key Data:** Provide any relevant studies, reports, or statistics.

- **Planning and decision making**: What is the process for planning of mental health services? What factors impact the decision-making process for service planning (i.e. politics, evidence-based decision making protocols, public interest, sustainability, availability of funding)? Is the planning consistent and transparent or ad-hoc?

- **Availability:** Are these services widely accessible or limited to certain areas?

- **Funding:** How are these services funded (e.g., government, private, donor-funded)?

- **Governance and cooperation:** How are these services governed? How are they integrated into the existing network of services, and how do they collaborate with one another?

- **Quality and Evaluation:** What is known about the quality of these services? Are there formal evaluations? (Are services monitored and evaluated for: feasibility and acceptability, need and accessibility, service-user evaluations, fidelity and impact evaluations, cost-effectiveness)

- **Gaps or Challenges:** Highlight any major gaps, barriers, or challenges faced in this area.

- **Sustainability and scalability**: Are the services sustainable in the long-term (consider trends political will, financing, human resources, coverage, access)? What are key barriers in scalability (substantial increase of availability and coverage services) in your country?

**Description (up to 500 words):**

**SWOT:**

a) Strengths

b) Weaknesses

c) Opportunities

d) Threats

### 4.2. Informal Community Care

Describe all activities which fall under the WHO definition of self-care below ([definition](#bookmark=id.lp0tu6x46zbm)).

In your description, include:

- **General Overview:** What services or programs exist?

- **Key Data:** Provide any relevant studies, reports, or statistics.

- **Planning and decision making**: What is the process for planning of mental health services? What factors impact the decision-making process for service planning (i.e. politics, evidence-based decision making protocols, public interest, availability of funding)? Is the planning consistent and transparent or ad-hoc?

- **Availability:** Are these services widely accessible or limited to certain areas?

- **Funding:** How are these services funded (e.g., government, private, donor-funded)?

- **Governance and cooperation:** How are these services governed? How are they integrated into the existing network of services, and how do they collaborate with one another?

- **Quality and Evaluation:** What is known about the quality of these services? Are there formal evaluations? (Are services monitored and evaluated for: feasibility and acceptability, need and accessibility, service-user evaluations, fidelity and impact evaluations, cost-effectiveness)

- **Gaps or Challenges:** Highlight any major gaps, barriers, or challenges faced in this area.

- **Sustainability and scalability**: Are the services sustainable in the long-term (consider trends political will, financing, human resources, coverage, access)? What are key barriers in scalability (substantial increase of availability and coverage services) in your country?

**Description (up to 500 words):**

**SWOT:**

a) Strengths

b) Weaknesses

c) Opportunities

d) Threats

### 4.3. Primary Care Mental Health Services

Describe all activities which fall under the WHO definition of primary care mental health services below ([definition](#bookmark=id.m30z10bruzte)).

In your description, include:

- **General Overview:** What services or programs exist?

- **Key Data:** Provide any relevant studies, reports, or statistics.

- **Planning and decision making**: What is the process for planning of mental health services? What factors impact the decision-making process for service planning (i.e. politics, evidence-based decision making protocols, public interest, availability of funding)? Is the planning consistent and transparent or ad-hoc?

- **Availability:** Are these services widely accessible or limited to certain areas?

- **Funding:** How are these services funded (e.g., government, private, donor-funded)?

- **Governance and cooperation:** How are these services governed? How are they integrated into the existing network of services, and how do they collaborate with one another?

- **Quality and Evaluation:** What is known about the quality of these services? Are there formal evaluations? (Are services monitored and evaluated for: feasibility and acceptability, need and accessibility, service-user evaluations, fidelity and impact evaluations, cost-effectiveness)

- **Gaps or Challenges:** Highlight any major gaps, barriers, or challenges faced in this area.

- **Sustainability and scalability**: Are the services sustainable in the long-term (consider trends political will, financing, human resources, coverage, access)? What are key barriers in scalability (substantial increase of availability and coverage services) in your country?

**Description (up to 500 words)**

**SWOT:**

a) Strengths

b) Weaknesses

c) Opportunities

d) Threats

### 4.4. Community Mental Health Services

Describe all activities which fall under the WHO definition of crisis services below ([definition](#bookmark=id.n5ocobonfw12)).

**a) Crisis Services**

- **General Overview:** What services or programs exist?

- **Key Data:** Provide any relevant studies, reports, or statistics.

- **Planning and decision making**: What is the process for planning of mental health services? What factors impact the decision-making process for service planning (i.e. politics, evidence-based decision making protocols, public interest, availability of funding)? Is the planning consistent and transparent or ad-hoc?

- **Availability:** Are these services widely accessible or limited to certain areas?

- **Funding:** How are these services funded (e.g., government, private, donor-funded)?

- **Governance and cooperation:** How are these services governed? How are they integrated into the existing network of services, and how do they collaborate with one another?

- **Quality and Evaluation:** What is known about the quality of these services? Are there formal evaluations? (Are services monitored and evaluated for: feasibility and acceptability, need and accessibility, service-user evaluations, fidelity and impact evaluations, cost-effectiveness)

- **Gaps or Challenges:** Highlight any major gaps, barriers, or challenges faced in this area.

- **Sustainability and scalability**: Are the services sustainable in the long-term (consider trends political will, financing, human resources, coverage, access)? What are key barriers in scalability (substantial increase of availability and coverage services) in your country?

**Description (up to 500 words)**

**SWOT:**

a) Strengths

b) Weaknesses

c) Opportunities

d) Threats

**b) Specialized Community Mental Health Services**

- **General Overview:** What services or programs exist?

- **Key Data:** Provide any relevant studies, reports, or statistics.

- **Planning and decision making**: What is the process for planning of mental health services? What factors impact the decision-making process for service planning (i.e. politics, evidence-based decision making protocols, public interest, availability of funding)? Is the planning consistent and transparent or ad-hoc?

- **Availability:** Are these services widely accessible or limited to certain areas?

- **Funding:** How are these services funded (e.g., government, private, donor-funded)?

- **Governance and cooperation:** How are these services governed? How are they integrated into the existing network of services, and how do they collaborate with one another?

- **Quality and Evaluation:** What is known about the quality of these services? Are there formal evaluations? (Are services monitored and evaluated for: feasibility and acceptability, need and accessibility, service-user evaluations, fidelity and impact evaluations, cost-effectiveness)

- **Gaps or Challenges:** Highlight any major gaps, barriers, or challenges faced in this area.

- **Sustainability and scalability**: Are the services sustainable in the long-term (consider trends political will, financing, human resources, coverage, access)? What are key barriers in scalability (substantial increase of availability and coverage services) in your country?

**Description (up to 500 words):**

**SWOT:**

a) Strengths

b) Weaknesses

c) Opportunities

d) Threats

### 4.5. Psychiatric services in general hospitals

Describe all activities which fall under the WHO definition of psychiatric services in general hospitals below ([definition](#bookmark=id.cjr4q97qz8s5)).

In your description, include:

- **General Overview:** What services or programs exist?

- **Key Data:** Provide any relevant studies, reports, or statistics.

- **Planning and decision making**: What is the process for planning of mental health services? What factors impact the decision-making process for service planning (i.e. politics, evidence-based decision making protocols, public interest, availability of funding)? Is the planning consistent and transparent or ad-hoc?

- **Availability:** Are these services widely accessible or limited to certain areas?

- **Funding:** How are these services funded (e.g., government, private, donor-funded)?

- **Governance and cooperation:** How are these services governed? How are they integrated into the existing network of services, and how do they collaborate with one another?

- **Quality and Evaluation:** What is known about the quality of these services? Are there formal evaluations? (Are services monitored and evaluated for: feasibility and acceptability, need and accessibility, service-user evaluations, fidelity and impact evaluations, cost-effectiveness)

- **Gaps or Challenges:** Highlight any major gaps, barriers, or challenges faced in this area.

- **Sustainability and scalability**: Are the services sustainable in the long-term (consider trends political will, financing, human resources, coverage, access)? What are key barriers in scalability (substantial increase of availability and coverage services) in your country?

**Description (up to 500 words):**

**SWOT:**

a) Strengths

b) Weaknesses

c) Opportunities

d) Threats

### 4.6. Long-stay facilities and specialist services

Describe all activities which fall under the WHO definition of long-stay facilities and specialist services in general hospitals below ([definition](#bookmark=id.ri0ijgp2xnlc)).

In your description, include:

- **General Overview:** What services or programs exist?

- **Key Data:** Provide any relevant studies, reports, or statistics.

- **Planning and decision making**: What is the process for planning of mental health services? What factors impact the decision-making process for service planning (i.e. politics, evidence-based decision making protocols, public interest, availability of funding)? Is the planning consistent and transparent or ad-hoc?

- **Availability:** Are these services widely accessible or limited to certain areas?

- **Funding:** How are these services funded (e.g., government, private, donor-funded)?

- **Governance and cooperation:** How are these services governed? How are they integrated into the existing network of services, and how do they collaborate with one another?

- **Quality and Evaluation:** What is known about the quality of these services? Are there formal evaluations? (Are services monitored and evaluated for: feasibility and acceptability, need and accessibility, service-user evaluations, fidelity and impact evaluations, cost-effectiveness)

- **Gaps or Challenges:** Highlight any major gaps, barriers, or challenges faced in this area.

- **Sustainability and scalability**: Are the services sustainable in the long-term (consider trends political will, financing, human resources, coverage, access)? What are key barriers in scalability (substantial increase of availability and coverage services) in your country?

**Description (up to 500 words):**

**SWOT:**

a) Strengths

b) Weaknesses

c) Opportunities

d) Threats

## Section 5: Mental health promotion and prevention

Describe all mental health prevention and promotion activities. This includes all non-clinical activities targeting prevention, promotion of mental well-being or low-intensity treatments for non-clinical populations.

Prevention

- Universal prevention: targeting the general public or a whole population group.
- Selective prevention: targeting individuals or subgroups of the population whose risk of developing a mental disorder is significantly higher than that of the rest of the population.
- Indicated prevention: targeting persons at high-risk for mental disorders.

Promotion

Any services aiming to facilitate populations in “the process of enabling people to increase control over, and to improve their health” (WHO, 1986)

1. **What is the current state of mental health promotion and prevention in your country? (self-harm & suicide, addiction, SMI, CMD, mental health promotion & well-being initiatives)**

- General overview
- Key programs and initiatives
- Key organizations responsible for policies and planning
- Key organizations responsible for implementation
- Key organizations responsible for monitoring and evaluation
- Key funders of prevention and promotion activities

**2. Are mental health promotion and prevention programs integrated into school facilities? If so, how?**

- Types of programs available
- Level of evidence of interventions
- Key implementing professionals
- Successes and challenges

**3. What initiatives exist for perinatal mental health promotion and prevention?**

- Availability of services
- Effectiveness and gaps

**4. Are there any early detection and early intervention services for people at risk of psychosis?**

- Yes/No
- If yes, please describe

**5. How is mental health promotion addressed in the workplace?**

- Key programs or policies
- Challenges in implementation

**6. What parenting or carers programs exist to support mental health?**

- Description of programs
- Effectiveness and reach
- Platform of service delivery

**7. How is mental health care integrated into primary health care and social care systems?**

- Integration methods
- Successes and areas for improvement

**8. Are there any digital mental health promotion and prevention initiatives in your country?**

- Yes/No
- If yes, please describe

## Section 6: E-mental health initiatives

E-mental health initiatives are defined as online interventions, and apps focusing on mental health, teletherapy, online therapy, and chat therapy. In general, initiatives that are utilizing online methods to increase mental health well-being should be considered.

1. **What regulations govern e-mental health in your country?**

- Is there any legislation specific to e-health or e-mental health?
  - If yes, what are the key provisions of this legislation?
- Are any e-mental health services covered by health insurance or similar schemes?
  - If yes, which services are covered, and which are not?
- Are there any initiatives that are run by your country's Department of Health or other government institutions?
  - If yes, which institutions and what is the focus of their initiatives?
- Are any regulatory changes being considered at present?

1. **What e-mental health initiatives are currently available in your country?**

- Types of services provided
- Target populations
- Are there any ongoing discussions or recent developments regarding these services?
- What is the country of origin of the e-mental health initiatives that are available in your country?

1. **How effective are these e-mental health initiatives?**

- Feedback from users and providers (number of users, number of regular users, time spent in an app, satisfaction, usefulness, etc.)
- Measured outcomes (e.g., improvement in mental health, service reach, etc.)

1. **What are the main challenges in implementing e-mental health initiatives in your country?**

- Technological barriers (including computer literacy)
- User engagement and accessibility
- Regulatory and policy challenges

1. **Are there plans to expand or further develop e-mental health services?**

- Yes/No
  - If yes, please provide details.
